# A Macromolecule Reversing Antibiotic Resistance Phenotype and Repurposing Drugs as Potent Antibiotics

**DOI:** 10.1002/advs.202001374

**Published:** 2020-07-21

**Authors:** Xin Ding, Chuan Yang, Wilfried Moreira, Peiyan Yuan, Balamurugan Periaswamy, Paola Florez de Sessions, Huimin Zhao, Jeremy Tan, Ashlynn Lee, Kai Xun Ong, Nathaniel Park, Zhen Chang Liang, James L. Hedrick, Yi Yan Yang

**Affiliations:** ^1^ School of Pharmaceutical Sciences (Shenzhen) Sun Yat‐sen University Shenzhen 518107 China; ^2^ Institute of Bioengineering and Nanotechnology 31 Biopolis Way, The Nanos Singapore 138669 Singapore; ^3^ Singapore‐MIT Alliance for Research and Technology (SMART) 1 CREATE Way Singapore 138602 Singapore; ^4^ Genome Institute of Singapore 60 Biopolis Street, Genome Singapore 138672 Singapore; ^5^ IBM Almaden Research Center 650 Harry Road San Jose CA 95120 USA

**Keywords:** antibiotics, combination therapy, drug repurposing, macromolecules, reversal of antibiotic resistance phenotype

## Abstract

In order to mitigate antibiotic resistance, a new strategy to increase antibiotic potency and reverse drug resistance is needed. Herein, the translocation mechanism of an antimicrobial guanidinium‐functionalized polycarbonate is leveraged in combination with traditional antibiotics to afford a potent treatment for drug‐resistant bacteria. Particularly, this polymer–antibiotic combination approach reverses rifampicin resistance phenotype in *Acinetobacter baumannii* demonstrating a 2.5 × 10^5^‐fold reduction in minimum inhibitory concentration (MIC) and a 4096‐fold reduction in minimum bactericidal concentration (MBC). This approach also enables the repurposing of auranofin as an antibiotic against multidrug‐resistant (MDR) Gram‐negative bacteria with a 512‐fold MIC and 128‐fold MBC reduction, respectively. Finally, the in vivo efficacy of polymer–rifampicin combination is demonstrated in a MDR bacteremia mouse model. This combination approach lays foundational ground rules for a new class of antibiotic adjuvants capable of reversing drug resistance phenotype and repurposing drugs against MDR Gram‐negative bacteria.

## Graphical abstract

Guanidinium‐functionalized polycarbonates reverse antibiotic resistance phenotype and improve antibiotic potency via intracellular protein precipitation, gene binding, and subsequent reactive oxygen species generation. Importantly, the polymer allows for the effective repositioning of rifampicin and auranofin meant for Gram‐positive bacteria and arthritis, respectively, to be highly effective against multidrug‐resistant Gram‐negative bacteria both in vitro and in vivo.

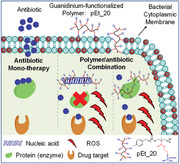

## 1 Introduction

The antibiotic development pipeline has run dry since the 1980s with few new antibiotics being approved for clinical use.^[^ ^1^ ^]^ Particularly, there is a serious lack of effective treatment options for drug‐resistant Gram‐negative bacterial infections. Carbapenem‐resistant Gram‐negative bacteria, including *Acinetobacter baumannii*, have been identified as the World Health Organization (WHO)’s critical priority pathogens for development of new treatment approaches.^[^ ^1^ ^]^ The surge of antibiotic resistance accompanied with the dearth of new antibiotics has made drug‐resistant bacterial infections a serious global threat,^[^ ^2^ ^]^ potentially claiming an estimated 10 million lives in 2050.^[^ ^3^ ^]^ To treat multidrug‐resistant (MDR) bacterial infections (especially Gram‐negative bacteria), the monotherapy of current antibiotics may no longer be adequate. Even for polymyxin antibiotics (polymyxin B and colistin), the last‐resort treatment for MDR Gram‐negative bacterial infections, resistance to them has been increasingly found in clinical isolates.^[^ ^4^ ^]^ As an alternative approach, combination therapy has attracted great attention. Although both positive and negative clinical outcomes were reported from the combination of different antibiotics for treatment of Gram‐negative bacterial infections,^[^ ^5^ ^]^ combining antibiotics with non‐antibiotic adjuvants has seen success in overcoming resistance against *β*‐lactams (e.g., piperacillin/tazobactam, amoxicillin/clavulanic acid, and meropenem/aspergillomarasmine A).^[^ ^6^ ^]^ In addition, antibiotic combinations with a food additive (vanillin/spectinomycin), with a membrane disruptor (colistin/macrolides), or a non‐antibiotic drug (procaine/doxycycline) were recently reported to afford a synergistic effect against MDR Gram‐negative bacteria.^[^ ^7^ ^]^ Colistin/macrolide combinations were active even at low concentrations in colistin‐resistant bacteria, although the precise mechanism underlying the synergistic effect is unclear.^[^ ^7^ ^]^ Among the adjuvants reported in the literature, membrane‐disruptive antimicrobials such as colistin have been extensively explored to potentiate antibiotic activity.^[^ ^7^, ^8^, ^9^, ^10^ ^]^ Despite this success, it is not advisable to use this last‐resort drug against MDR Gram‐negative bacteria as an antibiotic adjuvant as it may increase the incidence of colistin resistance.^[^ ^4^ ^]^ Moreover, no difference in clinical response rates between treatments with colistin and colistin/antibiotic combinations was observed for MDR Gram‐negative bacterial infections,^[^ ^11^, ^12^, ^13^, ^14^, ^15^ ^]^ and nephrotoxicity was increased with the combination therapy.^[^ ^12^ ^]^ Apart from colistin, several cationic antimicrobial polymers were also utilized as adjuvants to enhance antibacterial activity of antibiotics.^[^ ^16^, ^17^, ^18^ ^]^ Like colistin, these cationic polymers killed bacteria by membrane disruption. Therefore, they may face similar challenges as colistin/antibiotic combination has. Hence, antibiotic adjuvants with the ability to reverse antibiotic resistance phenotype enhance antibiotic potency and repurpose drugs as potent antibiotics are greatly needed.

**Figure 1:**
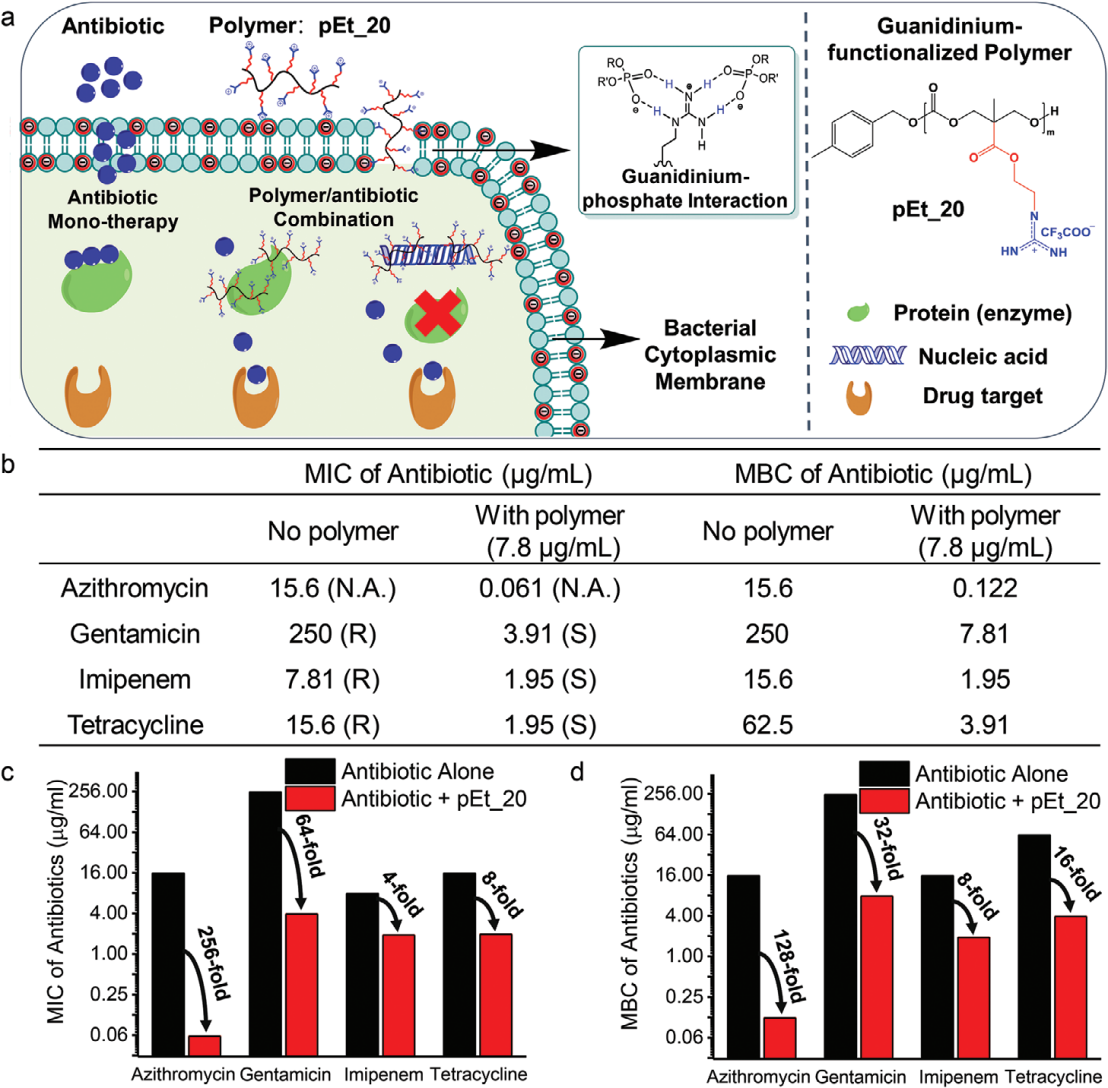
The polymer pEt_20 reverses antibiotic resistance phenotype in MDR Gram‐negative *A. baumannii* (BAA‐1789). a) Schematic presentation of the unique antimicrobial mechanism of the polymer–membrane penetration followed by binding of cytosolic proteins and genes, which might be a reason for reversing antibiotic resistance phenotype and enhancing antimicrobial activity. b) MICs and MBCs of antibiotics with and without pEt_20. MICs of antibiotics were interpreted according to the Performance Standards for Antimicrobial Susceptibility Testing of Clinical and Laboratory Standards Institute (CLSI), 2105. R: resistant; I: intermediate; S: susceptible. c) MIC fold reduction. d) MBC fold reduction of antibiotics in the presence of pEt_20. The concentration of pEt_20: 7.8 µg mL^−1^ (0.5× MIC), at which it did not kill bacteria (≈0% killing efficiency as compared to CFU at 0 h). The polymer pEt_20 reduced antibiotic MIC to the susceptible level or even lower, and enhanced bactericidal effect of the antibiotics as evidenced by decreased MBC values. Limit of detection: 50 CFU mL^−1^. MIC and MBC data are representatives of three biological replicates.

Polycarbonates are biodegradable polymers that have been studied for biomedical applications such as drug/gene delivery^[^ ^19^, ^20^, ^21^, ^22^, ^23^, ^24^, ^25^, ^26^, ^27^ ^]^ and antimicrobial materials^[^ ^28^, ^29^, ^30^ ^]^ due to their good biocompatibility and ease of incorporating various functional groups. Recently, we reported broad‐spectrum antimicrobial guanidinium‐functionalized polycarbonates with unique mechanism: membrane translocation followed by precipitation of cytosolic materials.^[^ ^31^ ^]^ Multiple treatments with the polymer neither increased their effective dose nor upregulated expression of genes associated with resistance as evidenced by RNA sequencing. Given the distinctive mechanism of the guanidinium‐based polycarbonate, we hypothesized that it could provide unique opportunities to reverse antibiotic resistance phenotype and enhance antimicrobial potency of the antibiotic as it could block the activity of antibiotic‐modifying genes or proteins and thereby increase the therapeutic effectiveness of the antibiotic in MDR infections (**Figure** **1**a).

## 2 Results and Discussion

### 2.1 Macromolecule Reverses Antibiotic Resistance Phenotype

We evaluated the potential of the guanidinium‐functionalized polycarbonate pEt_20 with degree of polymerization (DP) of 20 (Scheme S1, Supporting Information) to sensitize MDR bacteria to antibiotics having different antimicrobial mechanisms of action. In the MDR *A. baumannii* strain (BAA‐1789), which is resistant to all antibiotics tested (Figure 1b), the polymer mitigated the resistance against azithromycin, gentamicin, imipenem and tetracycline, and reduced their minimum inhibitory concentrations (MICs) to the susceptible level or even lower than the susceptible level (Figure 1b,c). The sensitization effect of the polymer was also reflected by reduction in the minimum bactericidal concentrations (MBCs) of the antibiotics (128‐, 32‐, 8‐, and 16‐fold reduction for azithromycin, gentamicin, imipenem, and tetracycline, respectively) (Figure 1b,d), where the polymer was used at 0.5× MIC (7.8 µg mL^−1^), which did not lead to a significant bactericidal effect (≈0% killing efficiency as compared to CFU at 0 h). These antibiotics kill bacteria by either inhibiting cell wall synthesis through interacting with penicillin binding proteins (imipenem) or preventing protein synthesis through binding 50S (azithromycin) or 30S subunit (tetracycline and gentamicin) of the ribosome. Imipenem resistance is mainly caused by carbapenemases that degrade *β*‐lactam,^[^ ^32^, ^33^, ^34^ ^]^ azithromycin resistance by methylase that alters the ribosomal target,^[^ ^35^ ^]^ tetracycline resistance by overexpression of ribosomal protection proteins that remove tetracycline from the ribosome,^[^ ^36^ ^]^ and gentamicin resistance by aminoglycoside‐modifying enzymes.^[^ ^37^, ^38^ ^]^ A possible reason for reversal of antibiotic resistance phenotype and sensitization of the MDR bacteria toward antibiotic treatment is the polymer's ability in nonspecific binding to cytosolic enzymes (proteins) or genes, including those that are responsible for antibiotic resistance.

Importantly, the polymer is capable of potentiating last‐line antibiotic colistin activity and reversing colistin‐resistance phenotype. Recently, plasmid‐mediated colistin‐resistance was reported in Gram‐negative bacteria, which resulted from MCR‐1 enzyme that catalyzes the transfer of phosphoethanolamine to lipid A, rendering the membrane more electropositive.^[^ ^39^, ^40^ ^]^ The MIC of colistin (colistimethate sodium) against two MCR‐1‐positive *Escherichia coli* strains was determined to be 15.6 and 31.3 µg mL^−1^, respectively (Table S1, Supporting Information), higher than the breakpoints of the Clinical and Laboratory Standards Institute (CLSI) standard for susceptibility testing (4–8 µg mL^−1^ for Gram‐negative bacteria). The polymer at 0.5× MIC (7.81 µg mL^−1^) significantly lowered colistimethate sodium MIC from 15.6–31.3 to 0.49 µg mL^−1^ (32‐ and 64‐fold reduction, respectively). More importantly, the MBC of colistimethate sodium was reduced by eightfold from 15.6 and 31.3 µg mL^−1^ to 1.95 and 3.91 µg mL^−1^, respectively, in the presence of the polymer where the polymer did not have a significant bactericidal effect (≈0% killing efficiency as compared to CFU at 0 h). The results demonstrate that the polymer sensitized the MCR‐1 colistin‐resistant bacteria to colistin treatment.

### 2.2 Macromolecule Repurposes Drugs

The polymer repurposed both the antituberculosis drug rifampicin and the antirheumatic drug auranofin, which are less effective against Gram‐negative bacteria than Gram‐positive bacteria (MIC of rifampicin: 1.95–31.3 µg mL^−1^ against Gram‐negative bacteria vs < 0.031 µg mL^−1^ for Gram‐positive *Staphylococcus aureus*; MIC of auranofin: 15.6–62.5 µg mL^−1^ against Gram‐negative bacteria vs 0.06–0.5 µg mL^−1^ for *S. aureus* ^[^ ^41^, ^42^ ^]^), as antibiotics with strong potency against Gram‐negative *A. baumannii* BAA‐1709 (Figure S1, Supporting Information). Rifampicin acts by binding the *β* subunit of RNA polymerase, thus inhibiting RNA synthesis,^[^ ^43^ ^]^ whereas auranofin inhibits thioredoxin reductase (TrxR), which disrupts thiol‐redox (Trx‐TrxR) balance essential in Gram‐positive bacteria as they lack the redox couple glutathione (GSH) and GSH reductase (GR), thus compromising defense against reactive oxygen species (ROS).^[^ ^42^ ^]^ However, GSH‐GR exists in many Gram‐negative bacteria, and thus Trx‐TrxR balance is not critical as evidenced by the high MIC values against Gram‐negative bacteria. In combination with the polymer, both rifampicin and auranofin showed for the first time a high level of activity against Gram‐negative bacteria. MICs of rifampicin and auranofin were reduced by 2048‐ and 128‐fold, respectively, against *A. baumannii* in combination with the polymer at 7.8 µg mL^−1^ (0.5× MIC) (Figure S1a,b, Supporting Information). Moreover, pEt_20 at 7.8 µg mL^−1^, where pEt_20 alone did not kill the bacteria (≈0% killing efficiency as compared to CFU at 0 h), significantly enhanced bactericidal activity of the drugs, and MBCs of rifampicin and auranofin were reduced from 3.91 and 15.6 µg mL^−1^ to 0.015 (256‐fold reduction for rifampicin) and 0.244 µg mL^−1^ (64‐fold reduction for auranofin) against *A. baumannii* (BAA‐1709), respectively (Figure S1b,c, Supporting Information).

**Figure 2:**
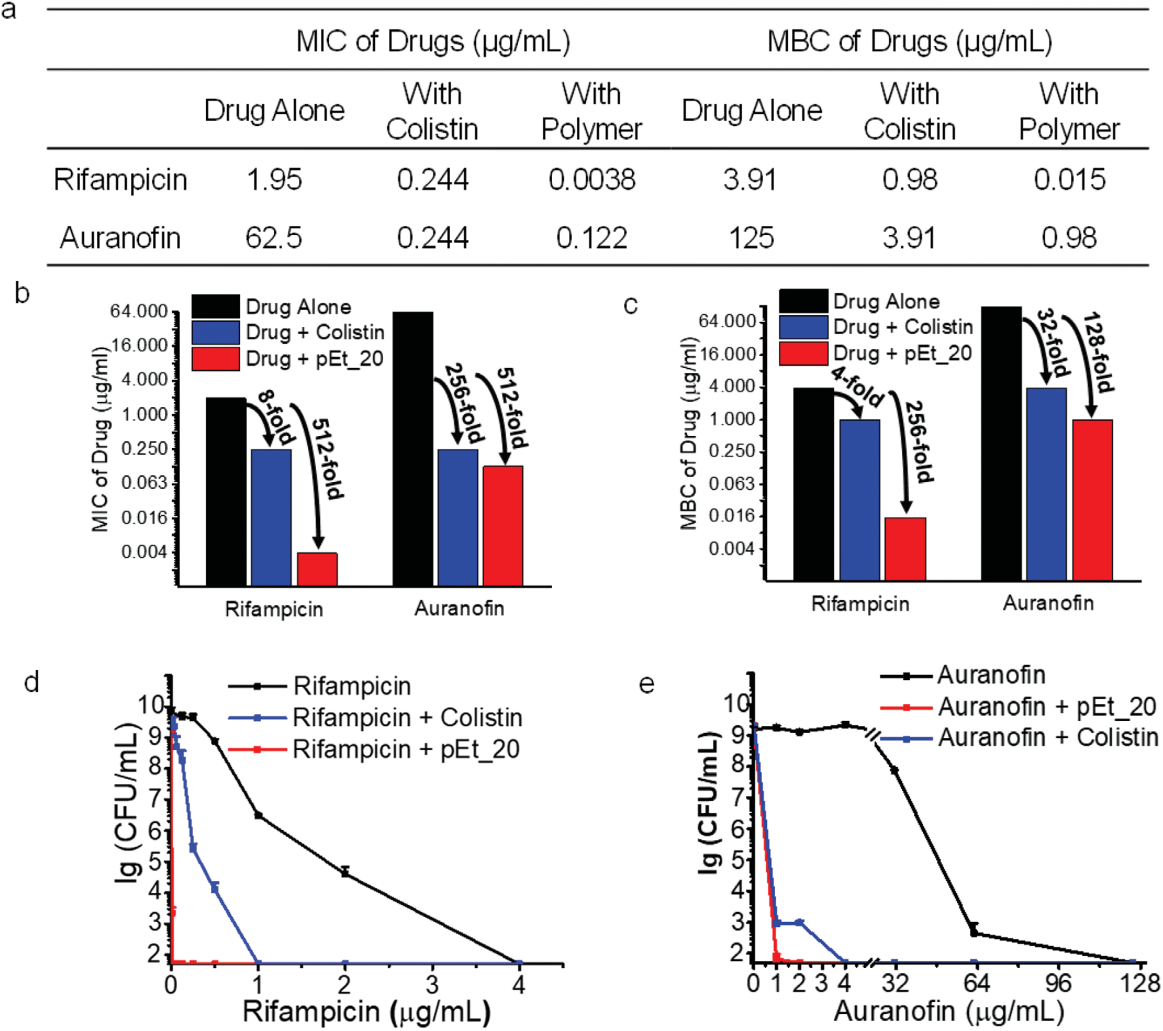
The polymer pEt_20 potentiates rifampicin and auranofin as potent antibiotics against Gram‐negative MDR *A. baumannii* (ATCC BAA‐1789). Rifampicin and auranofin are used in clinic for treatment of tuberculosis and rheumatoid arthritis, respectively. a) MICs and MBCs of rifampicin and auranofin with and without pEt_20. b) MIC folds reduction. c) MBC folds reduction of rifampicin and auranofin in the presence of pEt_20 in comparison with colistin sulfate. d) Killing efficiency of rifampicin and auranofin in the presence of pEt_20 in comparison with colistin sulfate. The polymer pEt_20 potentiated the antimicrobial activity of rifampicin and auranofin more effectively than colistin sulfate, leading to much greater MIC and MBC reduction (512‐ vs 8‐fold reduction in MIC and 256‐ vs 4‐fold reduction in MBC for rifampicin. 512‐ vs 256‐fold reduction in MIC and 128‐ vs 32‐fold reduction in MBC for auranofin). The pEt_20 combinations showed a stronger bactericidal effect (≥99.9% killing efficiency) than colistin sulfate. The concentration of pEt_20 and colistin sulfate: 7.8 and 0.5 µg mL^−1^ (0.5× MIC), respectively, at which, both pEt_20 and colistin sulfate did not kill bacteria (≈0% killing efficiency as compared to CFU at 0 h). Limit of detection: 50 CFU mL^−1^. MIC and MBC data are representatives of three biological replicates, and killing efficiency is presented as mean ± S.D. (*n* = 3).

**Figure 3:**
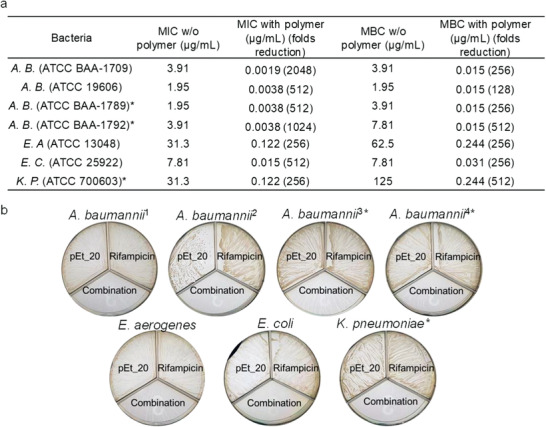
The polymer pEt_20 potentiates rifampicin in various types of Gram‐negative bacteria including MDR strains (*E. coli*, E. C., *E. aerogenes*, E. A., *K. pneumoniae*, K. P., and *A. baumannii*, A. B.). * Symbol indicates MDR strains. a) MICs and MBCs of rifampicin with and without polymer. The use of pEt_20 led to 256‐ to 2048‐fold and 128‐ to 512‐fold reduction in rifampicin MIC and MBC, respectively. pEt_20: 7.8 µg mL^−1^ (0.5× MIC). b) Three‐compartment plates with bacteria treated by pEt_20, rifampicin or pEt_20/rifampicin combination for 18 h. 1) *A. baumannii* (ATCC BAA‐1709), 2) *A. baumannii* (ATCC 179 606), 3) *A. baumannii* (ATCC BAA‐1789), 4) *A. baumannii* (ATCC BAA‐1792). The combination was bactericidal (≥99.9% killing efficiency) against all bacterial strains tested, while pEt_20 or rifampicin did not kill the bacteria (≈0% killing efficiency as compared to that at 0 h) when used alone. No bacterial colonies were seen in the groups treated by the combination. The concentration of pEt_20 in (b) 0.5× MIC, i.e., 7.8 µg mL^−1^ for all A. B. strains and E. C., 15.6 µg mL^−1^ for E. A. and K. P. MIC and MBC data are representative of three biological replicates.

Moreover, the polymer/rifampicin and polymer/auranofin combinations showed strong potency against MDR *A. baumannii* (**Figure** **2**), where MIC dropped by 512‐fold for both rifampicin and auranofin in the presence of pEt_20 at 7.8 µg mL^−1^ (0.5× MIC) (Figure 2a,b). The polymer reduced MBC of rifampicin and auranofin by 256‐ and 128‐fold, respectively (Figure 2a,c–e). A similar phenomenon was also seen for the polymer/rifampicin combination at a polymer concentration of 0.5× MIC in other types of Gram‐negative bacteria, including *Enterobacter aerogenes*, *E. coli*, MDR *Klebsiella pneumoniae*, and other *A. baumannii* strains, with MIC reduction between 256‐ and 2048‐fold (**Figure** **3**a). In addition, the polymer at 0.5× MIC, where no bactericidal activity was observed (≈0% killing efficiency as compared to CFU at 0 h), reduced the MBC of rifampicin by 128‐ to 512‐fold (Figure 3a,b). Moreover, the polymer at 0.5× MIC reduced MIC of rifampicin derivatives (rifaximin and rifabutin) by 512‐fold against *A. baumannii* (BAA‐1709) (Table S2, Supporting Information). We postulate that binding of the polymer with cytosolic genes or proteins is in part responsible for its ability of potentiating the antimicrobial activity of rifampicin and its derivatives. In the mechanistic study presented later, we demonstrated that polymer treatment increased intracellular ROS of the bacteria. Hence, removal of bacterial defense by auranofin against ROS can explain for the enhanced killing of the bacteria.

### 2.3 Stark Difference between Membrane Translocation and Membrane Lytic Macromolecules

Although the use of colistin (colistin sulfate) and quaternary ammonium‐based polycarbonate (Qua_20, Scheme S1, Supporting Information),^[^ ^44^ ^]^ which kill bacteria by membrane disruption, reduced rifampicin MIC, guanidinium‐based polycarbonates (pEt_10, pEt_20, and pEt_40, with DP of 10, 20, and 40, respectively) (Scheme S1, Supporting Information) potentiated rifampicin more effectively, reducing rifampicin MIC by 2048‐ to 4096‐fold at a polymer concentration of 0.5× MIC against *A. baumannii* (BAA‐1709) as compared to 16‐, 4‐, and 4‐fold reduction for colistin sulfate, Qua_20 and polyarginine (R_10_), respectively (Table S3, Supporting Information). The presence of the polymers with different lengths led to similar MIC of rifampicin, and the difference in the MIC is ≤2 times. This is considered no significant difference as MIC was measured by the commonly used twofold serial dilution method.^[^ ^31^ ^]^ Therefore, the molecular weight of the polymer did not affect the antibacterial activity of rifampicin. In addition, pEt_20 potentiated auranofin more effectively than colistin sulfate with greater MIC and MBC fold reduction: 128‐ versus 8‐fold MIC reduction and 64‐ versus 2‐fold MBC reduction for the drug‐susceptible *A. baumannii* (BAA‐1709) (Figure S1, Supporting Information), 512‐ versus 256‐fold MIC reduction and 128‐ versus 32‐fold MBC reduction for MDR *A. baumannii* (BAA‐1789) (Figure 2). In the presence of pEt_20, a lower concentration of rifampicin (Figure 2d and Figure S1d, Supporting Information) or auranofin (Figure 2e and Figure S1e, Supporting Information) was required to kill the same number of MDR *A. baumannii* (BAA‐1789) or *A. baumannii* (BAA‐1709) as compared to colistin sulfate, which is a typical membrane‐lytic antibiotic. Moreover, the polymer was more effective than colistin sulfate in reducing MIC and MBC of azithromycin against both strains: 8‐ versus 2‐fold MIC reduction and 16‐ versus 4‐fold MBC reduction for the drug‐susceptible strain (Figure S2, Supporting Information), 256‐ versus 16‐fold MIC reduction (Figure 1b) and 128‐ versus 4‐fold MBC reduction for the MDR strain (Figure S2, Supporting Information). The overall superiority of the polymer/antibiotic combination over colistin sulfate/antibiotic combination was further demonstrated by faster killing efficiency. With colistin sulfate, a longer duration of time is required to kill a similar number of bacteria (Figure S3, Supporting Information).

### 2.4 Macromolecule Reverses Rifampicin Resistance Phenotype in Mutants

**Figure 4:**
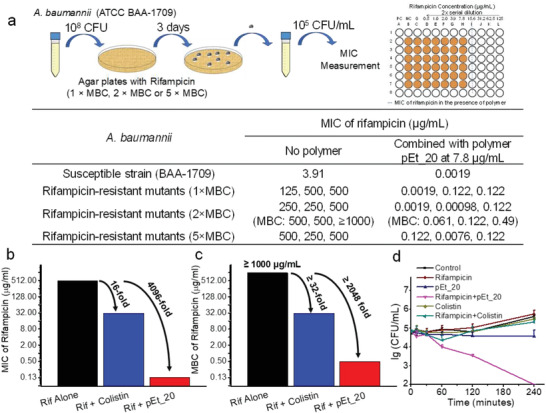
The combination of pEt_20 and rifampicin reverses rifampicin resistance phenotype developed in *A. baumannii* (BAA‐1709) mutants. a) The rifampicin‐resistant mutants were selected by culturing the bacteria (≈10^8^ CFUs) on agar containing rifampicin at 1×, 2× and 5× MBC, i.e., 3.9, 7.8, and 19.5 µg mL^−1^, respectively) for three days. The table shows tremendous MIC and MBC elevation for all the three mutants at each concentration, while the polymer at 0.5× MIC (i.e., 7.8 µg mL^−1^) reversed rifampicin resistance phenotype in the mutants, lowering MIC and MBC by up to ≈2.5 × 10^5^‐fold. At 7.8 µg mL^−1^, the polymer did not exert any bactericidal effect (≈0% killing efficiency as compared to CFU at 0 h). b) Fold reduction in rifampicin MIC. c) Fold reduction in rifampicin MBC against a mutant developed with rifampicin at 2× MBC in the presence of pEt_20 and colistin sulfate at their 0.5× MIC (i.e., 7.8 and 0.5 µg mL^−1^). Although colistin sulfate reduced rifampicin MIC and MBC in the mutants, it is much less effective than pEt_20. d) Killing kinetics of rifampicin (0.5× MIC against *A. baumannii* 1709, 2.0 µg mL^−1^), pEt_20 (0.5× MIC against both *A. baumannii* 1709 and rifampicin‐resistant *A. baumannii* 1709 mutants, 7.8 µg mL^−1^), pEt_20/rifampicin combination (pEt_20: 7.8 µg mL^−1^, rifampicin: 2.0 µg mL^−1^), colistin sulfate (0.5× MIC against both *A. baumannii* 1709 and rifampicin‐resistant *A. baumannii* 1709 mutants, 0.50 µg mL^−1^) and colistin sulfate/rifampicin combination (colistin sulfate: 0.50 µg mL^−1^, rifampicin: 2.0 µg mL^−1^) against rifampicin‐resistant mutants (developed from 2× MBC). Combination of pEt_20 and rifampicin killed rifampicin‐resistant *A. baumannii* 1709 (3 log reduction in bacteria count in 4 h), while the combination with colistin sulfate did not show any bactericidal activity (≈0% killing efficiency as compared to CFU at 0 h). Limit of detection: 100 CFU mL^−1^. The data are representative of three biological replicates.

To further study if the use of the macromolecule is able to reverse antibiotic resistance, rifampicin‐resistant *A. baumannii* mutants were developed by inoculating 10^8^ CFUs on an agar plate containing different lethal concentrations of rifampicin (MBC, 2× MBC and 5× MBC) (**Figure** **4**a). A number of *A. baumannii* mutants were developed after 3 days of incubation even at 5× MBC, and MIC of rifampicin increased from 3.9 to 125–500 µg mL^−1^ against the mutants. Whole genome sequencing of the evolved strains at the three concentrations (three colonies for every concentration) was performed. All nine isolates independently acquired mutations in the *rpoB* gene, and no other mutation was observed relative to control strains that were subjected to the same assay conditions except for antibiotic treatment. Mutations in *rpoB* were reported with *A. baumannii* clinical isolates, which led to high MICs (≈256–512 µg mL^−1^),^[^ ^45^ ^]^ similar to what we observed with our in vitro evolved rifampicin‐resistant colonies. In sharp contrast, no resistant colonies to the polymer were found on the plates even when the inoculum increased from 10^8^ to 10^9^ CFUs, implying that the frequency of resistance to the polymer was less than 2.1 × 10^−10^. More importantly, the polymer reversed the rifampicin resistance phenotype of the mutants, reducing MIC of rifampicin to 0.00098–0.122 µg mL^−1^ (up to 2.5 × 10^5^‐fold reduction) (Figure 4a). In addition, pEt_20 at 0.5× MIC, where there was no significant bactericidal effect (≈0% killing efficiency as compared to CFU at 0 h), decreased the MBC of rifampicin from 500 to 0.122 µg mL^−1^ (4096‐fold reduction) against one of the mutants developed with rifampicin at 2× MBC (Figure 4b), which is even lower than rifampicin MBC against the parent strain (3.9 µg mL^−1^) (Figure 3a), further demonstrating its ability of reversing rifampicin resistance phenotype. Although colistin sulfate also reduced rifampicin MIC and MBC in the mutants (Figure 4b), it is 256‐fold (MIC) and 64‐fold (MBC) less effective than the polymer (16‐ vs 4096‐fold reduction in rifampicin MIC and ≥32‐ vs ≥2048‐fold reduction in rifampicin MBC for colistin sulfate and polymer, respectively) (Figure 4b,c). It is worth noting that colistin sulfate is unable to reverse rifampicin resistance phenotype in the mutants as MIC and MBC of rifampicin against the mutants in the presence of colistin sulfate at 0.5× MIC (i.e., 0.5 µg mL^−1^) were still higher than those of rifampicin against the parent strain (MIC and MBC: 31.3 vs 3.91 µg mL^−1^) (Figure 4b,c). Moreover, polymer/rifampicin combination showed a bactericidal effect (99.9% killing efficiency) against the mutants within 4 h, while colistin sulfate/rifampicin combination did not kill the mutants within the same time frame (Figure 4d). These findings demonstrate that pEt_20 reverses rifampicin resistance phenotype, and enhances the antimicrobial activity of rifampicin against the mutants much more effectively than colistin sulfate.

### 2.5 Mechanistic Study

**Figure 5:**
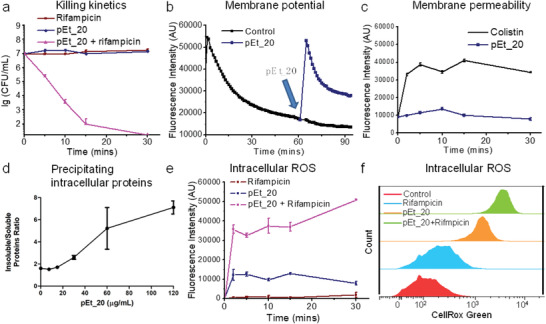
Mechanistic study of combination treatment using pEt_20 and rifampicin. a) Rapid killing kinetics of the combination (>99.9% killing in 10 min). pEt_20: 0.5× MIC (7.8 µg mL^−1^), rifampicin: 0.5× MIC (0.50 µg mL^−1^). Limit of detection: 50 CFU mL^−1^. b) Analysis of membrane potential of *A. baumannii* 1709 using DiSC3, a cationic fluorescent dye that accumulates onto negatively charged bacterial membrane through electrostatic interaction, quenching fluorescence. pEt_20 interacted with the phosphate groups on the membrane through strong bidendate hydrogen‐bonding interaction, which released DiSC3, increasing fluorescence intensity. After membrane translocation of pEt_20^16^, the phosphate groups were released for binding DiSC3, decreasing fluorescence intensity. pEt_20: 7.8 µg mL^−1^. c) Fluorescence of bacteria treated with pEt_20 and PI dye, which only stained bacteria with damaged membrane. Colistin sulfate significantly increased membrane permeability, while pEt_20 did not exert a significant effect (pEt_20 and colistin sulfate at 0.5× MIC, 7.8 and 0.50 µg mL^−1^, respectively). This finding is in agreement with our previous study that the pEt_20 translocated membrane instead of lysing it.^[^ ^16^ ^]^ d) Soluble and insoluble protein quantification following polymer treatment. Log‐phase culture of *A. baumannii* 1709 was treated with increasing concentration of pEt_20 for 15 min, resulting in an increase in insoluble/soluble protein ratio. The polymer treatment caused bacterial protein precipitation in a dose‐dependent manner. Data shown are the results of two independent biological replicates carried out in technical triplicates. e) Fluorescence intensity analysis of intracellular ROS probe CellRox Green in *A. baumannii* BAA‐1709 (≈10^7^ CFU mL^−1^) after treatment with rifampicin (0.50 µg mL^−1^), pEt_20 (7.8 µg mL^−1^), and their combination (pEt_20: 7.8 µg mL^−1^; rifampicin: 0.50 µg mL^−1^) over various periods of time. f) Flow cytometry histograms. The results from (e,f) show that the combination significantly enhanced intracellular ROS generation. This might be responsible for the rapid bacteria killing of the combination under the same treatment conditions in a. pEt_20 translocated bacterial membrane followed by binding cytosolic proteins or genes (Figure S4, Supporting Information), facilitating ROS generation and thus killing the bacteria.

We next explored the general antimicrobial mechanism of the polymer/antibiotic combination using polymer/rifampicin as an example. Membrane potential and permeability of *A. baumannii* was studied after polymer treatment at a sublethal dose that did not kill the bacteria during the experimental period of 30 min (≈0% killing efficiency as compared to CFU at 0 h) (**Figure** **5**a). Polymer treatment resulted in transient membrane depolarization. Specifically, the membrane potential was reduced upon bidendate hydrogen‐bonding interaction between guanidinium groups in the polymer and anionic phosphate groups on the membrane,^[^ ^46^ ^]^ but it then increased gradually after membrane translocation of the polymer (Figure 5b). Alexa‐Fluor‐488‐labeled polymer was seen in the membrane and cytosol of the bacterial cells after translocation (Figure S4a, Supporting Information). Contrary to membrane‐lytic colistin sulfate, polymer treatment caused minimal membrane disruption (Figure 5c). These findings further suggest that unlike colistin sulfate, the polymer does not exert its antibacterial synergistic effect via membrane disruption or permeabilization but rather via a membrane translocation mechanism that enables its interaction with essential cytosolic biomacromolecules of the bacterial cell. As shown in Figure 5d, the polymer treatment resulted in a significant increase in the amount of insoluble proteins in the bacteria as reflected by the increase of insoluble to soluble protein ratio. Additionally, pEt_20 effectively bound bacterial DNA and inhibited DNA mobility (Figure S4b, Supporting Information). We predicted that this unique mechanism of action of polymer pEt_20 would translate to an overwhelming cytosolic stress, which would mediate rapid bacterial cell death. As shown in Figure 5e,f and Figures S5–S7 in the Supporting Information, polymer treatment led to significant production of intracellular ROS of both drug‐susceptible *A. baumannii* BAA‐1709 and rifampicin‐resistant *A. baumannii* BAA‐1709 mutant (developed from 2× MBC of rifampicin, i.e., 7.8 µg mL^−1^) within a few minutes even at a sublethal dose (0.5× MIC). The amount of ROS increased substantially when polymer/rifampicin combination was used. The increase in the ROS level might be due to greater cytosolic stress caused by the complementary functional mechanisms of polymer (binding with nucleic acids) and rifampicin (inhibition of nucleic acid synthesis). DNA binding by antimicrobial polymers was also observed by Zhou et al.^[^ ^47^ ^]^ to cause strong SOS response, leading to elevation of intracellular ROS level.^[^ ^48^ ^]^ The rapid production of ROS implies a final effector role in bacterial cell death following treatment with polymer/rifampicin combination.^[^ ^49^ ^]^

The overwhelming stress experienced by the bacteria undergoing treatment with the combination therapy was also supported by the findings from next generation RNA‐seq analysis (Figure S8, Supporting Information). Treatment of *A. baumannii* BAA‐1709 with the combination therapy for 5 min led to the upregulation of various genes associated with stress response pathways, including acyl‐CoA dehydrogenase^[^ ^50^ ^]^ and NADH dehydrogenase,^[^ ^51^ ^]^ which catalyze ROS production, heat shock proteins,^[^ ^52^ ^]^ phage‐like and bacteriophage genes,^[^ ^53^ ^]^ as well as NADH‐quinone oxidoreductase^[^ ^54^ ^]^ (Tables S4–S7, Supporting Information). This phenomenon was not observed in the bacteria treated with either rifampicin or polymer alone. This further indicates that the combination therapy was highly potent, resulting in massive stress experienced by the bacteria. Interestingly, the gene associated with bacteriolytic activity (bacteriolytic lipoprotein entericidin B) was upregulated only in the bacteria treated with pEt_20/rifampicin combination, suggesting that the combination therapy might trigger programmed cell death.^[^ ^55^ ^]^

### 2.6 Toxicity Evaluation and In Vivo Antimicrobial Efficacy

To evaluate if the polymer/rifampicin combination is suitable for in vivo application, in vitro and in vivo toxicity was investigated. Rifampicin, polymer and their combination did not cause significant hemolysis in rat red blood cells (rRBCs) even at 500 µg mL^−1^ (Figure S9a, Supporting Information), and the combination did not increase cytotoxicity of rifampicin in HEK293T human embryonic kidney cell line (Figure S9b, Supporting Information). In addition, the polymer likely does not have the tendency to interact with genetic materials of mammalian cells as it did not enter the nucleus at least after 1 h (Figure S9c, Supporting Information), which is desirable for future clinical application. Moreover, similar to rifampicin, liver and kidney functions, sodium ion and potassium ion concentrations remained largely unchanged at 2 and 14 days post treatment with the combination (Figure S10a, Supporting Information). The combination therapy did not cause liver and kidney tissue damage (Figure S10b,c, Supporting Information). These findings demonstrate that the combination treatment did not induce in vivo toxicity.

**Figure 6:**
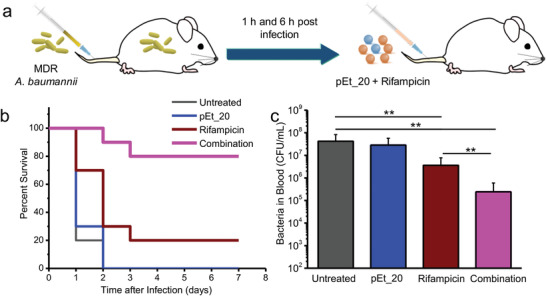
In vivo synergistic antimicrobial effects of pEt_20/rifampicin combination in a mouse bacteremia model. a) The mouse bacteremia model was created by injection of MDR *A. baumannii* (ATCC BAA‐1789) at 1.3 × 10^9^ CFU mL^−1^ (200 µL/20 g). pEt_20 (2.0 mg kg^−1^) and rifampicin (5.0 mg kg^−1^) were sequentially injected into mouse tail vein with two doses at 1 and 6 h post infection (*n* = 10). b) Survival of the infected mice after different treatments. The combination therapy provided a significantly higher survival rate than monotherapy using rifampicin or pEt_20. c) Blood bacterial counts from MDR *A. baumannii* (6.5 × 10^8^ CFU mL^−1^)‐infected mice at 24 h post infection. The combination treatment led to 2.4 log CFU reduction, which is significantly higher than rifampicin monotherapy with 1.1 log CFU reduction. (Means ± S.D. *n* = 6. One‐way ANOVA (Tukey's post hoc); **p < 0.01.)

After demonstrating in vivo applicability of the combination, in vivo antimicrobial efficacy of the combination was evaluated in a mouse model of bacteremia caused by MDR *A. baumannii* (BAA‐1789) (**Figure** **6**a). Importantly, the rifampicin/polymer combination therapy provided a significantly higher survival rate than monotherapy using rifampicin or polymer (Figure 6b). An increased rifampicin dose in the combination led to higher survival rates (Figure S10d, Supporting Information). Similarly, the combination therapy reduced the blood bacterial load more effectively than rifampicin (2.4‐log CFU reduction vs 1.1‐log CFU reduction in the bacterial counts) (Figure 6c). These results were achieved with a single day treatment.

## 3 Conclusion

We have demonstrated that the co‐delivery of polymer with antibiotics reverses antibiotic resistance phenotype, and improves antibiotic potency. In addition, the use of the polymer allows for the effective repositioning of rifampicin and auranofin meant for Gram‐positive bacteria and arthritis, respectively, to be highly effective against MDR Gram‐negative bacteria. Phenotypic analysis has demonstrated the affinity of pEt_20 to the membrane, transient membrane depolarization, gene binding, intracellular protein precipitation, and subsequent ROS generation. Polymer/rifampicin combination leads to a significantly enhanced level of intracellular ROS that subsequently causes bacterial death. The translation of this combination therapy has been successfully demonstrated in a MDR *A. baumannii*‐caused bacteremia mouse model. This broadly applicable therapeutic combination approach with distinctive mechanisms shows great promise in the treatment of MDR Gram‐negative bacterial infections.

## 4 Experimental Section

##### Materials

*A. baumannii* strains BAA‐1709, 19606, BAA‐1789 (MDR strain), BAA‐1792 (MDR strain), *S. aureus* strain 6538, *E. coli* strain 25922, *E. aerogenes* 13048*, K. pneumonia* 700603, and *Pseudomonas aeruginosa* strain 9027 were purchased from American Type Culture Collection (ATCC). The antibiotics rifampicin, tetracycline, azithromycin, ciprofloxacin, ceftazidime, and colistin sulfate were bought from MedChem Express. Colistimethate sodium was purchased from Cayman Chemical. Penicillin G was obtained from Sigma‐Aldrich. Gentamicin was purchased from Gold Biotechnology. Polymyxin B was bought from Merck. Imipenem was obtained from MerckSharp & Dohme Corp. Peptide R_10_ (RRRRRRRRRR‐CONH2) was bought from GL Biochem (Shanghai). MM 4–64 dye (*N*‐(3‐triethylammoniumpropyl)‐4‐(6‐(4‐(diethylamino)phenyl)hexatrienyl)pyridinium dibromide) was purchased from Santa Cruz. SYTOX Green was purchased from Life Technologies.

##### Polymer Synthesis and Characterization

Guanidinium‐functionalized polycarbonates (pEt_10, pEt_20, and pEt_40)^[^ ^31^ ^]^ and Alexa‐Fluor‐488‐labeled pEt_20^[^ ^31^ ^]^ as well as quaternary ammonium‐functionalized polycarbonate (Qua_20)^[^ ^44^ ^]^ were synthesized by advanced organocatalytic living ring‐opening polymerization and characterized according to the previously reported protocols. All polymers had well‐defined length and narrow molecular weight distribution (polydispersity index: 1.1–1.2).

##### MIC and MBC Measurements

To evaluate the antibacterial activity of antibiotics in the presence of polymer or colistin sulfate, the broth microdilution method was employed to measure MIC.^[^ ^56^ ^]^ Bacteria suspension (*A. baumannii*, *E. coli, E. aerogenes*, or *K. pneumoniae*), which grew overnight was diluted with MHB to ≈10^5^ CFU mL^−1^. 96‐well plates containing diluted bacteria suspension and serially diluted antibiotics, polymer, colistin sulfate, or their combinations were incubated at 37 °C with shaking at 100 rpm for 18–20 h. The concentration, at which there was no bacterial growth reflected by unchanged optical density (OD) at 600 nm measured using a microplate reader (TECAN, Switzerland), was recorded as MIC. To evaluate the bactericidal activity of antibiotics, polymer, colistin sulfate, or their combinations, the bacteria suspension after 18 h treatment with antibiotics, polymer, colistin sulfate, or their combinations was serially diluted and streaked on LB agar plates. MBC was determined by counting the colonies formed on the plates after overnight incubation at 37 °C. The concentration of antibiotics, polymer, or colistin sulfate, at which there was more than 99.9% bacterial colony reduction, was recorded as MBC. MIC and MBC experiments were conducted in triplicates.

##### Killing Efficiency and Killing Kinetics

*A. baumannii* BAA‐1709 (≈10^5^ CFU mL^−1^) was incubated with antibiotic, pEt_20, colistin sulfate, pEt_20/antibiotic combination or colistin/antibiotic combination at their 0.5× MIC (pEt_20:7.8 µg mL^−1^ and colistin sulfate: 0.5 µg mL^−1^). After incubation at 37 °C with shaking at 100 rpm, the bacteria were collected at 18 h and then serially diluted. The diluted bacterial suspension (20 µL) was streaked onto LB agar plates and CFUs were counted after overnight incubation at 37 °C. The results of CFU counting are presented as mean ± SD of three samples. In addition, the killing kinetics of rifampicin alone, rifampicin in combination with pEt_20 or colistin sulfate at their 0.5× MIC was also tested. Specifically, *A. baumannii* BAA‐1709 (≈10^5^ CFU mL^−1^) was incubated with rifampicin alone (0.016 µg mL^−1^) and rifampicin in combination with pEt_20 or colistin sulfate at their 0.5× MIC (pEt_20: 7.8 µg mL^−1^; colistin sulfate: 0.5 µg mL^−1^). At predetermined time points (10, 30 min, 1, 2, 4, 24 h), a small portion of the bacterial suspension was collected, serial diluted, and streaked on LB agar plates. After incubation overnight, CFUs were counted and the results were presented as mean ± SD of three samples.

##### Mutant Selection

*A. baumannii* BAA‐1709 (≈10^8^ CFU) were streaked onto MHB agar plates which contained rifampicin at concentrations of 1× MBC (3.9 µg mL^−1^), 2× MBC (7.8 µg mL^−1^), or 5× MBC (39.0 µg mL^−1^) (three replicates for each concentration). The bacteria on the plates were incubated at 37 °C for 3 days. The colonies appearing on the plates were restreaked on rifampicin‐containing agar plates to confirm their resistance. The resistant colonies were collected and stored at −80 °C for MIC and MBC measurements of rifampicin in the presence of pEt_20 or colistin sulfate.

In addition, to explore the antimicrobial mechanism of the polymer, mutant selection was also performed using pEt_20. Specifically, *A. baumannii* BAA‐1709 (≈10^8^ CFU) were streaked on MHB agar plates which contained pEt_20 at concentrations of 1× MBC_(agar)_ (750 µg mL^−1^). *A. baumannii* BAA‐1709 (≈10^6^ CFU) were applied onto MHB agar containing pEt_20 at different concentrations, and the concentration with at least 99.9% CFU reduction was determined to be the MBC_(agar)_. However, no resistant colonies were found after 3‐day incubation. Moreover, there were no resistant colonies detected even when the bacteria loading was increased to 1.6 × 10^9^ CFU. The results indicate that the frequency of resistance to pEt_20 was < 1/(3 × 1.6 × 10^9^) = 2.1 × 10^−10^ (3 represents triplicates).

##### Membrane Permeability

*A. baumannii* BAA‐1709 cells (≈10^7^ CUF mL^−1^) were incubated with colistin sulfate (0.5 µg mL^−1^, 0.5× MIC) or pEt_20 (7.8 µg mL^−1^, 0.5× MIC) for 2, 5, 10, 15, and 30 min. The cells were washed with phosphate‐buffered saline (PBS) and PI dye (final concentration: 2 µg mL^−1^) was added. After 30 min incubation, 200 µL of bacterial suspension was added into a black 96‐well plate and the fluorescence intensity (Ex: 535 nm, Em: 620 nm) was recorded by using a Tecan Spark 10M microplate reader.

##### Membrane Potential

*A. baumannii* BAA‐1709 cells were diluted to ≈10^7^ CUF mL^−1^ with MHB medium. The potentiometric probe DiSC_3_(5) was added in the medium at a final concentration of 1 × 10^−6^ m. The fluorescence intensity (Ex: 620 nm, Em: 680 nm) of DiSC_3_(5) was monitored for 1 h in dark at room temperature (200 µL per well in black 96‐well plate). The polymer with final concentration at 7.8 µg mL^−1^ was added to the medium containing the bacteria and DiSC_3_(5), and the fluorescence intensity was monitored using the microplate reader.

##### Quantification of Intracellular Protein Precipitation

5 mL of *A. baumannii* BAA‐1709 log phase culture was treated with increasing concentration of pEt_20 for 24 h followed by centrifugation. Supernatant was discarded and cells were resuspended in 300 µL of PBS containing protease inhibitor cocktail. Bacterial cells were lysed in nondenaturing buffer (Tris‐HCL, 0.1 m) by bead‐beating followed by centrifugation. The supernatant (soluble fraction) was collected. 300 µL of PBS was added to residual volume. Beads were allowed to settle down for 1 h before 250 µL was collected (insoluble fraction). Both fractions were dosed using standard BCA assay.^[^ ^57^ ^]^

##### Gene Binding Assay

*A. baumannii* (ATCC BAA‐1709) was cultured in MHB medium overnight (100 rpm, 37 °C). The bacterial suspension (50 mL) was then centrifuged and the pellet was lysed by G2 buffer. The genomic DNA was extracted using an EZ1 DNA Tissue Kit (48) in the EZ1 machine (QIAGEN) following the EZ1 DNA Tissue protocols. The gene binding ability of pEt_20 was investigated by agarose gel electrophoresis. DNA extracted from *A. baumannii* BAA‐1709 was mixed with pEt_20 at various polymer to DNA mass ratios (1–20). Briefly, 9.5 µL of the pEt_20/gene complex solution containing 300 ng of gene and corresponding polymer at their respective mass ratios was mixed with 0.5 µL of 5×  DNA loading dye. The mixture (7 µL) was loaded into individual wells of 1% agarose gel containing SYBR Safe DNA Gel Stain (Thermofisher Scientific, USA) at a ratio of 1:10 000 1× TAE buffer. The same amount of naked DNA was used as the control. The gel was run at 120 mV for 20 min in 1× TAE buffer. Following completion of the assay, the gel was imaged using a gel imaging system fitted with a transilluminator (Bio‐rad, U.S.A.).

##### ROS Level

*A. baumannii* BAA‐1709 or rifampicin‐resistant *A. baumannii* BAA‐1709 mutant developed from 2× MBC of rifampicin (i.e., 7.8 µg mL^−1^) (≈0^7^ CUF mL^−1^) were incubated with rifampicin (0.5 µg mL^−1^), pEt_20 (7.8 µg mL^−1^, 0.5× MIC) and their combination for 2, 5, 10, 15, and 30 min. The bacterial cells were then washed with PBS. CellRox Green dye (final concentration: 2.5 × 10^−6^ m) was added into the bacterial suspension and incubated for 30 min at room temperature. Bacterial cells were washed three times with PBS and 200 µL of bacterial suspension was added into a black 96‐well plate for fluorescence measurement. The fluorescence intensity (Ex: 485 nm, Em: 528 nm) was recorded by using the microplate reader.

##### Flow Cytometry

The fluorescence level of CellRox Green was also evaluated by flow cytometry. The samples were prepared using the same protocol as described in the previous paragraph, and the treatment time with rifampicin, pEt_20 and their combination was 10 min. The bacteria after treatment were analyzed by using a flow cytometer (BD FACSCelesta).

##### Confocal Microscopic Study

Permeability of pEt_20 across *A. baumannii* BAA‐1709 cell membrane was studied using Alexa‐Fluor‐488‐labeled pEt_20 at sub‐MIC. The bacterial cell suspension at logarithmic growth phase was diluted to ≈10^7^ CUF mL^−1^ with MHB medium. The diluted bacterial suspension was incubated with the dye‐labeled polymer (7.8 µg mL^−1^, 0.5× MIC) for 30 min at 37 °C, and then washed once with PBS. The bacteria were stained with the blue nucleic acid dye Hoechst 33 342 1 µg mL^−1^ for 10 min and followed by staining with the red membrane dye MM4‐64 (5 µg mL^−1^) on ice for 1 min. The bacteria after staining were spun down at 3000 g for 2 min at 4 °C, followed by being resuspended in 50 µL of PBS. The bacteria suspension was applied onto glass cover slide, and then observed under a confocal microscope with a 100× objective lens (Zeiss LSM 5 DUO, Germany).

CellRox Green staining was performed to probe intracellular ROS. *A. baumannii* BAA‐1709 cells (≈10^7^ CUF mL^−1^) were incubated with pEt_20 (7.8 µg mL^−1^, 0.5× MIC), rifampicin (0.5 µg mL^−1^) or their combination for 10 min at 37 °C, and then washed once with PBS. The CellRox Green dye (final concentration: 2.5 × 10^−6^ m) was incubated with the treated bacteria for 30 min at room temperature, and then washed three times with PBS. Following the same protocol described in the previous paragraph, the Hoechst and MM4‐64 dye were used to stain the bacteria for observation under a confocal microscopy (Olympus FV1000).

##### RNA Extraction, Library Preparation, and RNA‐seq Pipeline

*A. baumannii* BAA‐1709 (≈10^8^ CFU mL^−1^) was incubated with pEt_20 (31.3 µg mL^−1^), rifampicin (7.8 µg mL^−1^), or the combination (pEt_20: 15.6 µg mL^−1^ and rifampicin: 0.5 µg mL^−1^) for 5 min at 37 °C. The total RNA of the treated bacteria was isolated by TRIzol reagent (Invitrogen) based on the suggested protocol, and the isolated RNA was purified using PureLink RNA mini kit (Invitrogen). RNA integrity was checked using Agilent 4200 TapeStation System. Samples were preprocessed using Ribo‐Zero rRNA removal kit for bacteria (Illumina) followed by library preparation using TruSeq Stranded mRNA Library Prep Kit (Illumina). Libraries were multiplexed and run on HiSeq 4000 Sequencing Systems (Illumina) to yield 2× 151 bp paired‐end reads.

RNA‐seq data (paired end FASTQ files, 2 × 151 bp) were mapped to the parent strain, *A. baumanii* SDF genome using the Subread aligner (subread package version 1.6.3).^[^ ^58^ ^]^ Gene level read counts were calculated using feature Counts (part of the Subread package).^[^ ^59^ ^]^ Gene level differential gene expression analysis was performed using Bioconductor package EdgeR.^[^ ^60^ ^]^ Initially, gene counts were transformed into counts per million values and only those genes that had cpm ≥1 across all samples were considered, followed by library size adjustment and trimmed mean of *M*‐values (TMM) normalization. Statistical significance was calculated using exact test (edgeR) and significantly differentially expressed genes were filtered based on False discovery rate ≤0.05 and log twofold change of at least plus or minus 1. Significant genes with at least a log twofold change of 1 are presented in Table S4 in the Supporting Information (rifampicin/control), Table S5 in the Supporting Information (pEt_20/control), and Table S6 in the Supporting Information (rifampicin and pEt_20 combination/control), respectively.

##### DNA Extraction, Library Preparation, and Whole Genome Sequencing Pipeline

Selective colonies from the agar plates (i.e., rifampicin‐resistant *A. baumannii* BAA‐1709 mutants) were cultured overnight. 1 mL of the culture was pelleted and treated with Proteinase K, and DNA extraction was carried out using the EZ1 DNA Extraction Kit on the EZ1 Advanced XL automated DNA purification machine (Qiagen Inc., Hilden, Germany). Library preparation was performed using NEBNext Ultra DNA Library Prep Kit (NEB Inc., Massachusetts, USA). Libraries were multiplexed and subjected to sequencing (paired end, 2 × 151 bp) on the Illumina HiSeq 4000 sequencing platform. Overall, an average per‐base coverage of ≈503× was obtained. Paired end FASTQ files were mapped using BWA‐MEM (version 0.7.10) against the parent *A. baumannii* SDF strain. Single nucleotide variants were called using LoFreq* (version 2.1.2).^[^ ^61^ ^]^ The same analysis procedure was followed for all in vitro evolved rifampicin‐resistant strains (three colonies/MBC concentration and three different MBC concentrations; nine colonies in total) and the corresponding control strains (three colonies) that were subjected to the same culture and plating conditions except for rifampicin treatment. The single nucleotide variants that appeared in the control strains were filtered from the resistant strains to identify the selective mutations that contributed to resistance. Mutation was observed only in *rpoB* gene for all nine evolved colonies. The raw FASTQ files of both controls and resistant clones were uploaded to NCBI SRA (BioProject accession: PRJNA543935).

##### Hemolytic Activity and Cytotoxicity Analysis

To analyze hemolytic activity, rRBCs were diluted 25‐fold in PBS to achieve 4% v/v of blood content and the rRBCs were treated with rifampicin alone or rifampicin in combination with pEt_20 (7.8 µg mL^−1^). The untreated rRBCs and 0.1% triton‐treated rRBCs were used as negative control and positive control, respectively. The absorbance at 576 was taken to reflect the hemolysis level.

MTT assay was employed to evaluate the cytotoxicity of rifampicin and pEt_20/rifampicin combination. Briefly, HEK 293 cells (10^4^ cells per well) were incubated with rifampicin or rifampicin in combination with pEt_20 (7.8 µg mL^−1^) for 24 h at 37 °C with 5% CO_2_. MTT reagent was added and incubated for 4 h, and the MTT reagent was removed and the wells were washed. Subsequently, dimethyl sulfoxide (DMSO) was added to dissolve the purple formazan crystals on the plates. The blank wells and untreated cells were used as negative control and positive control, respectively. The absorbance at 570 nm was taken to reflect cell viability. The results from both assays were calculated using the following formula: (absorbance_sample_ − absorbance_negative control_)/(absorbance_postitive control −_ absorbance_negative control_). Each assay was conducted at least in triplicates.

##### Animal Studies

Female ICR mice (ICR = Institute of Cancer Research) (6–8 weeks) were used for analysis of in vivo antimicrobial activity of rifampicin/pEt_20 combination. The experiments were conducted according to protocols approved by the Institutional Animal Care and Use Committee of Agency for Science, Technology and Research, Singapore. To establish a mouse bacteremia model, immunosuppressant cyclophosphamide (200 mg kg^−1^) was injected intraperitoneally 4 days prior to infection. The immunosuppressed mice were then infected with MDR *A. baumannii* (ATCC BAA‐1789) at 1.3 × 10^9^ CFU mL^−1^ (100 µL/10 g of mouse body weight) by intravenous (i.v.) injection via tail vein. At 1 and 6 h post infection, rifampicin (5 mg kg^−1^), polymer pEt_20 (2 mg kg^−1^), rifampicin/pEt_20 combination or PBS was administered to the infected mice through i.v. injection (ten mice per group). The mice were monitored for 14 days to plot Kaplan–Meier survival curves.

To further assess the bacteria reduction in blood and other organs, the blood infection was induced by i.v. injection of MDR *A. baumannii* (ATCC BAA‐1789) at 6.5 × 10^8^ CFU mL^−1^ (100 µL/10 g of mouse body weight). The same treatments (rifampicin at 5 mg kg^−1^, pEt_20 at 2 mg kg^−1^, rifampicin/pEt_20 combination or PBS) were given to the infected mice (six mice per group). At 24 h post infection, the blood (10 µL) was collected from tail vein, and the organs (heart, liver, spleen, and lung, liver) were harvested and homogenized. The blood and homogenate were then serially diluted and streaked on LB agar plates. After incubation overnight, the colonies were counted and the data were presented as mean ± SD (*n* = 6).

##### Systemic Toxicity Analysis

ICR mice (6–8 weeks) without infection were treated at the same doses as the treatments applied in the infection model (rifampicin at 5 mg kg^−1^), rifampicin/pEt_20 combination, or PBS). After 2 and 14 days, the blood (0.8–1.0 mL) was collected through cardiac puncture. Alanine transaminase (ALT), aspartate transaminase (AST), creatinine, urea nitrogen, sodium, and potassium ion levels in the blood were analyzed by IDEXX Catalyst One Chemistry Analyzer. Statistic difference between the untreated and rifampicin or rifampicin/pEt_20 combination groups were analyzed by One‐way ANOVA. At 14 days post infection and treatments, the mice were sacrificed and the liver and kidney tissues were harvested. The samples were fixed in 4% formalin and then stained with hematoxylin–eosin (H&E) using standard protocols. The optical images were obtained with a stereomicroscope (Nikon, USA).

## Conflict of Interest

The authors declare no conflict of interest.

## Supporting information

Supporting Information

Supplemental Table 4

Supplemental Table 5

Supplemental Table 6

Supplemental Table 7
