# Supplementary material for: A Macromolecule Reversing Antibiotic Resistance Phenotype and Repurposing Drugs as Potent Antibiotics
Source: Adv Sci (Weinh). 2020 Jul 21;7(17):2001374. doi: 10.1002/advs.202001374 (PMC7503100; doi:10.1002/advs.202001374)
Supplement: Supplementary file 1 — Supporting Information [file ADVS-7-2001374-s001.pdf]

Copyright WILEY-VCH Verlag GmbH & Co. KGaA, 69469 Weinheim, Germany, 2020.

Supporting Information

A macromolecule reversing antibiotic resistance phenotype and repurposing drugs as potent antibiotics

Xin Ding, Chuan Yang, Wilfried Moreira, Peiyan Yuan, Balamurugan Periaswamy, Paola Florez de Sessions, Huimin Zhao, Jeremy Tan, Ashlynn Lee, Kai Xun Ong, Nathaniel Park, Zhen Chang Liang, James L. Hedrick and Yi Yan Yang**

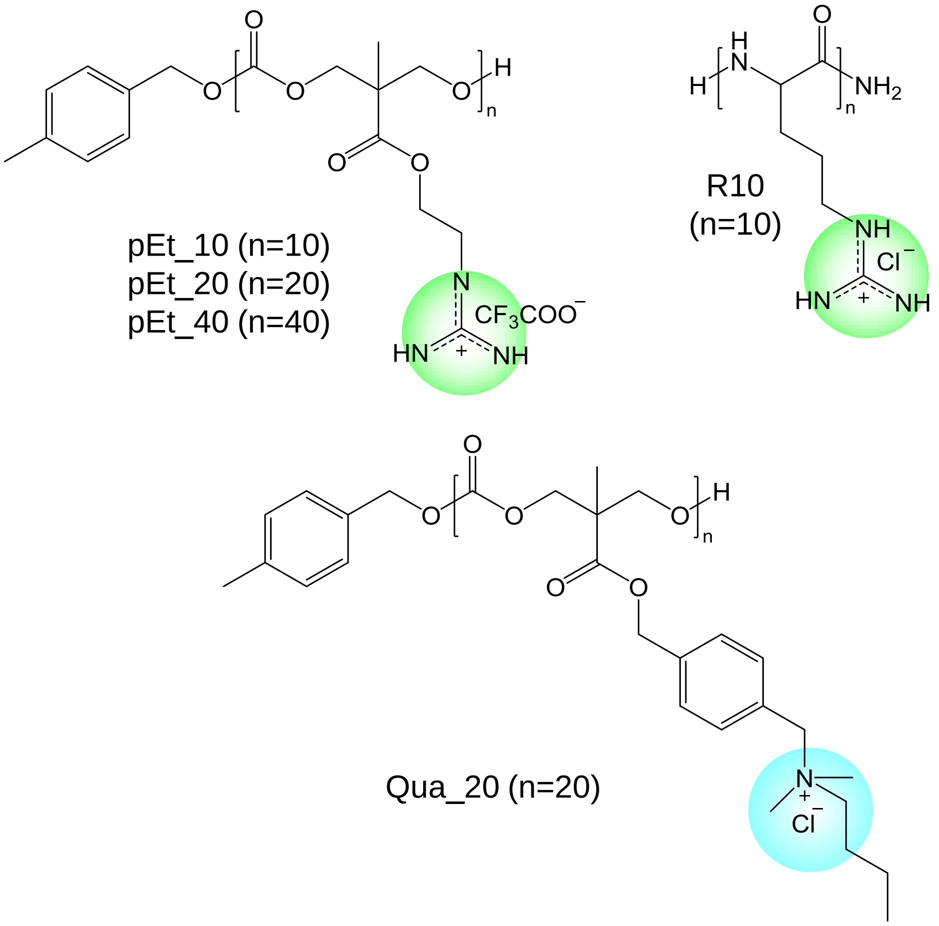

Scheme S1. Chemical structures of polymers used in this study.

a

	MIC of Drugs (µg/mL)			MBC of Drugs (µg/mL)		
	Drug Alone	With Colistin	With Polymer	Drug Alone	With Colistin	With Polymer
Rifampicin	3.91	0.12	0.0019	3.91	1.95	0.015
Auranofin	15.6	1.95	0.122	15.6	7.81	0.244

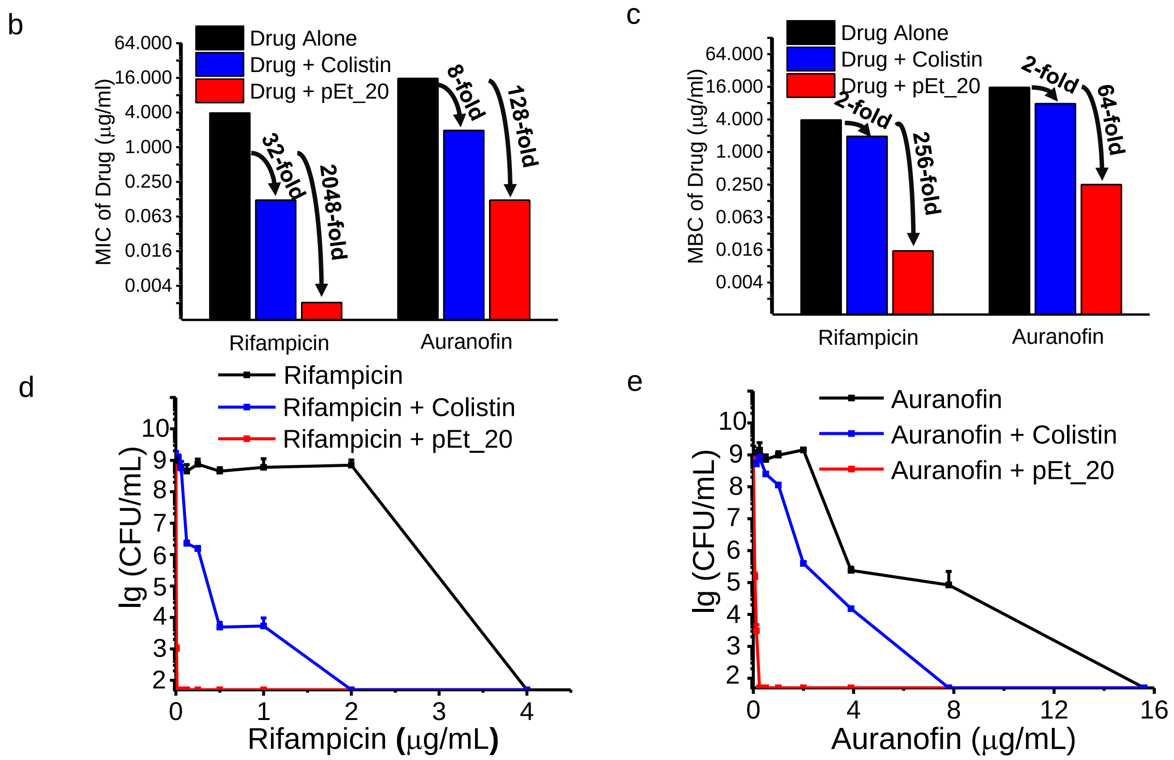

Figure S1. The polymer pEt_20 potentiates rifampicin and auranofin as potent antibiotics against drug-susceptible *A. baumannii* (BAA-1709). **a** MICs and **b** MBCs of rifampicin and auranofin with and without pEt_20 in comparison with colistin sulfate; **c** MIC fold change and **d** MBC fold change of rifampicin and auranofin in the presence of pEt_20 or colistin sulfate; **d** Killing efficiency of rifampicin and **e** killing efficiency of auranofin in the presence of pEt_20 in comparison with colistin sulfate. The polymer pEt_20 potentiated rifampicin and auranofin more effectively than colistin sulfate, leading to much greater MIC and MBC reduction (2048- *vs.* 32-fold reduction in MIC and 256- *vs.* 2-fold reduction in MBC for rifampicin; 128- *vs.* 8-fold reduction in MIC and 64- *vs.* 2-fold reduction in MBC for auranofin). The pEt_20 combinations showed a much stronger bactericidal effect (≥99.9% killing efficiency) than the colistin sulfate combinations. MIC and MBC data are representatives of three biological replicates, and killing efficiency is presented as Mean ± S.D. (n=3).

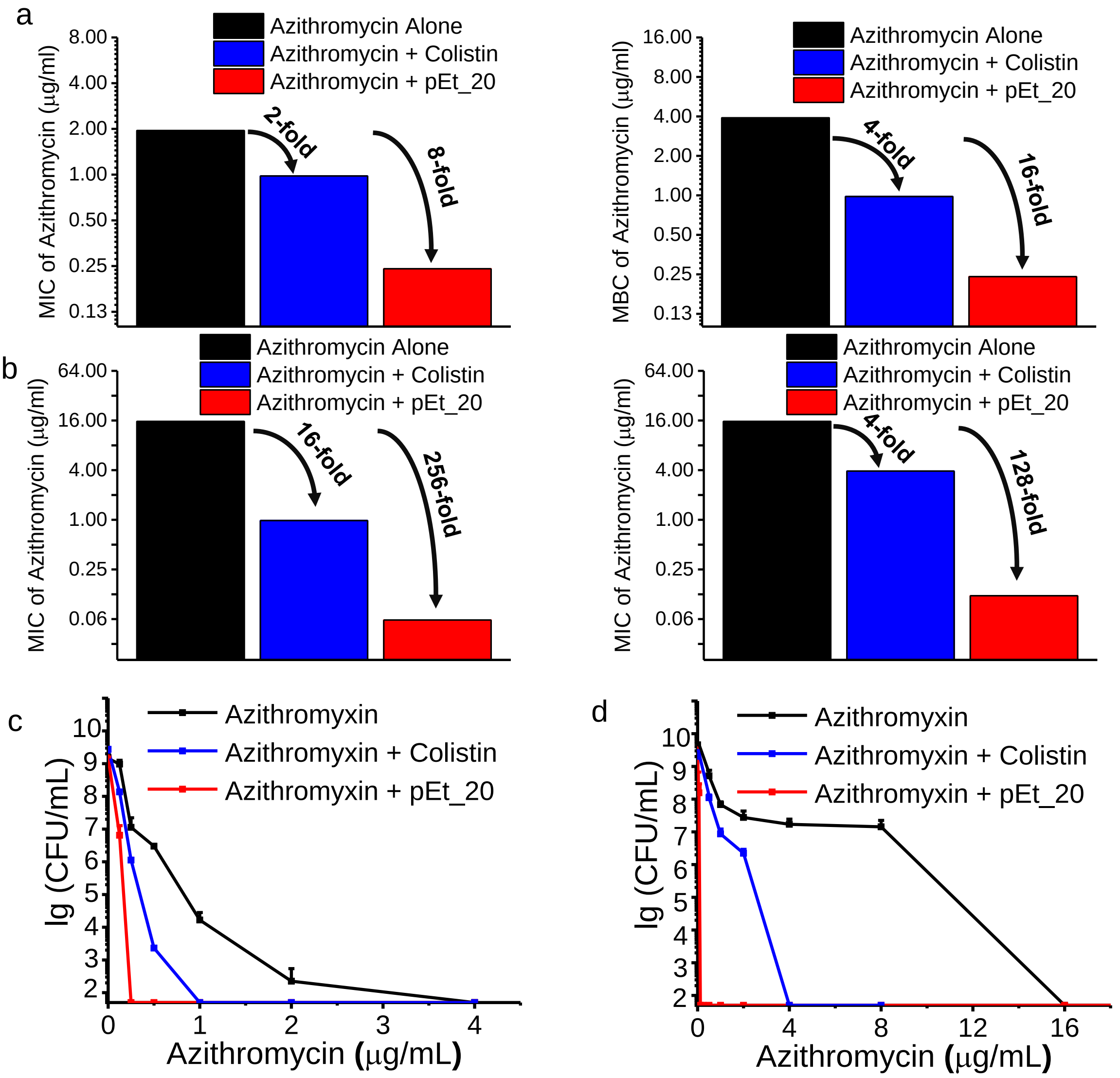

Figure S2. The polymer pEt_20 enhances antimicrobial activity of azithromycin against *A. baumannii* in comparison with colistin sulfate. **a** MIC and MBC of azithromycin against drug-susceptible *A. baumannii* (BAA-1709) and **b** MIC and MBC of azithromycin against MDR *A. baumannii* (BAA-1789) with or without pEt_20 in comparison with colistin sulfate; **c** Killing efficiency of azithromycin against *A. baumannii* (BAA-1709) and **d** Killing efficiency of azithromycin against *A. baumannii* (BAA-1789) with and without pEt_20 in comparison with colistin sulfate. The polymer pEt_20 reduced azithromycin MIC and MBC more effectively than colistin sulfate, and pEt_20/azithromycin combination showed a stronger bactericidal effect (≥99.9% killing efficiency) than colistin sulfate/azithromycin combination. MIC and MBC data are representatives of three biological replicates, and killing efficiency is presented as Mean ± S.D. (n=3).

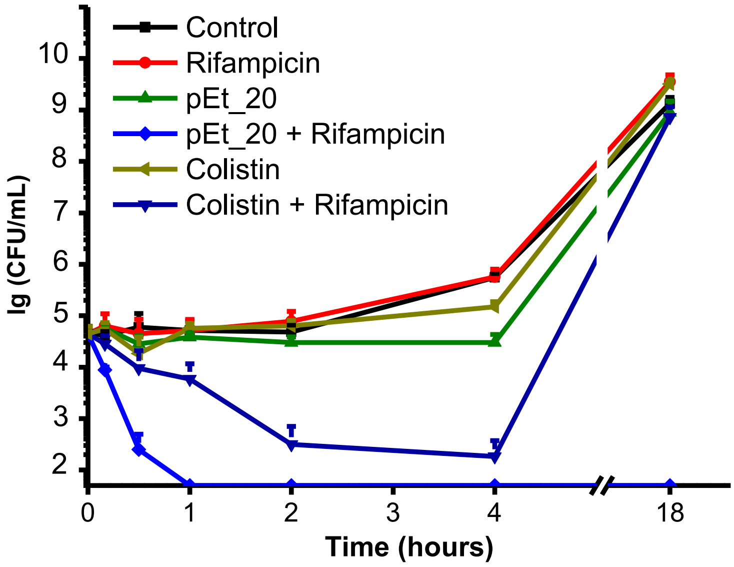

Figure S3. Killing kinetics of pEt_20 (½ × MIC, i.e. 7.8 µg/mL), rifampicin (0.015 µg/mL), pEt_20/rifampicin combination, colistin sulfate (½ × MIC, i.e. 0.50 µg/mL), and colistin sulfate /rifampicin combination against *A. baumannii* BAA-1709 (~10^5 CFU/mL). pEt_20/rifampicin combination killed the bacteria effectively within 1 h (3 log reduction in bacterial counts, i.e. >99.9% killing), while colistin sulfate /rifampicin combination did not show bactericidal activity at 18 h (~0% killing efficiency as compared to CFU at 0 h). Limit of detection: 50 CFU/mL. The data are representative of three biological replicates.

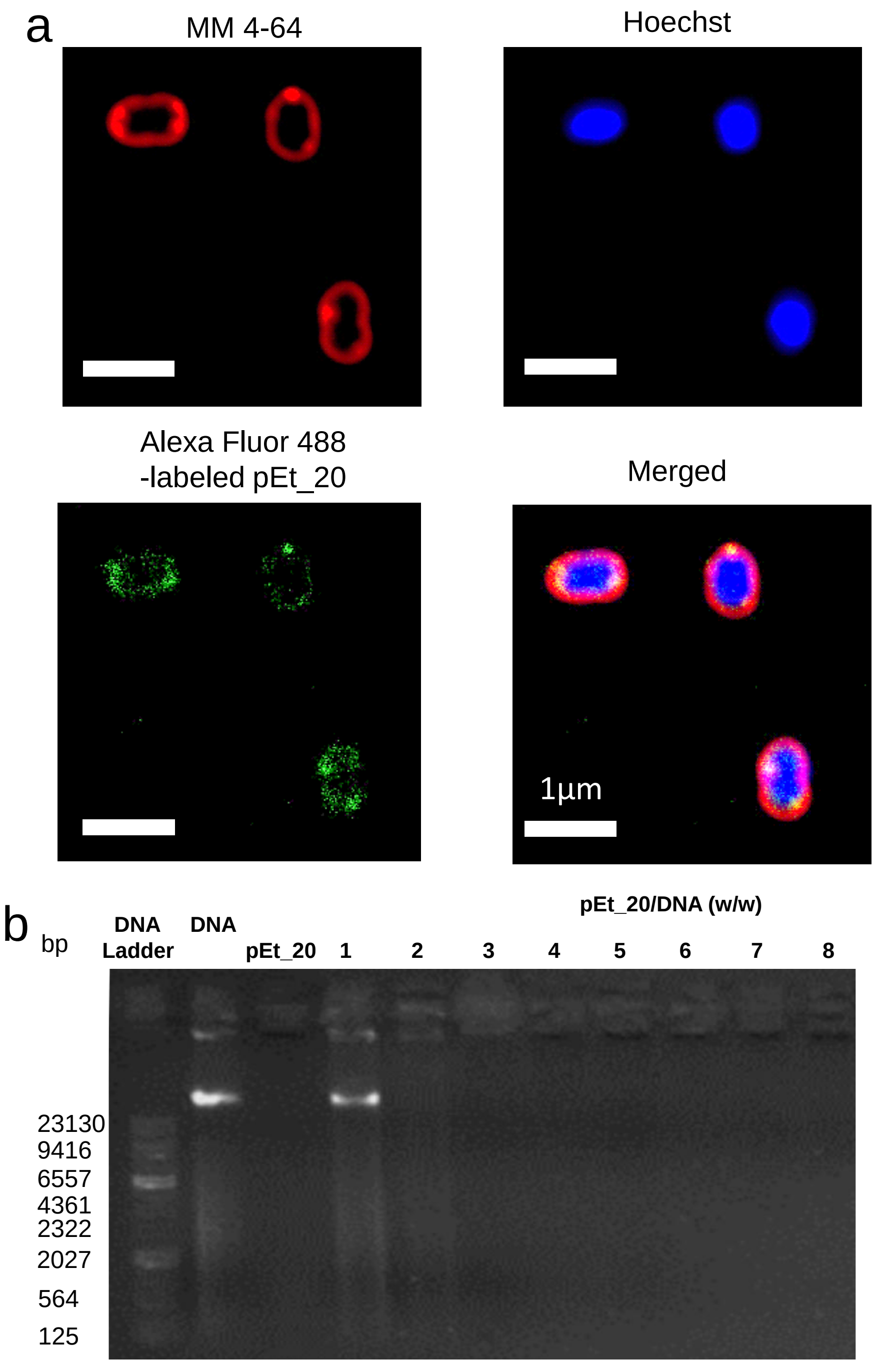

Figure S4. pEt_20 translocated bacterial membrane and bound with cytosolic DNA. **a** Confocal microscopic images of *A. baumannii* BAA-1709 after 30 min treatment with pEt_20 at 0.5×MIC (7.8 μg/mL) (Red regions: MM 4-64 stained membrane; Blue regions: Hoechst stained nucleic acids; Green regions: Alexa Fluor 488-labeled pEt_20); **b** Electrophoretic mobility of cytosolic DNA of *A. baumannii* BAA-1709 in pEt_20/DNA complexes at various weight ratios of polymer to DNA as specified. pEt_20 effectively bound DNA and completely inhibited the mobility of DNA at the polymer to DNA mass ratio of 3 and above.

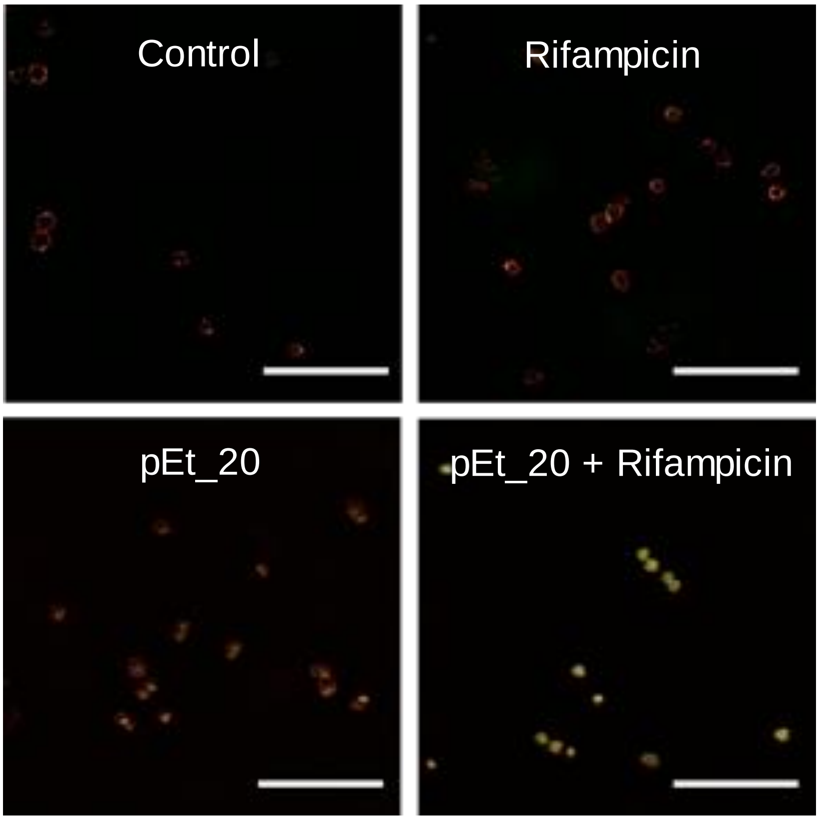

Figure S5. Increase in intracellular ROS of *A. baumannii* BAA-1709 after treatment with pEt_20 or pEt_20/rifampicin combination. Confocal microscopic analyses [Red dye (MM 4-64): membrane staining; Green dye (CellRox Green): ROS probe] of the bacteria before and after 10-min treatment with rifampicin (0.50 μg/mL), pEt_20 (7.8 μg/mL) and their combination (pEt_20: 7.8 μg/mL; rifampicin: 0.50 μg/mL). (Scale bar: 10 μm)

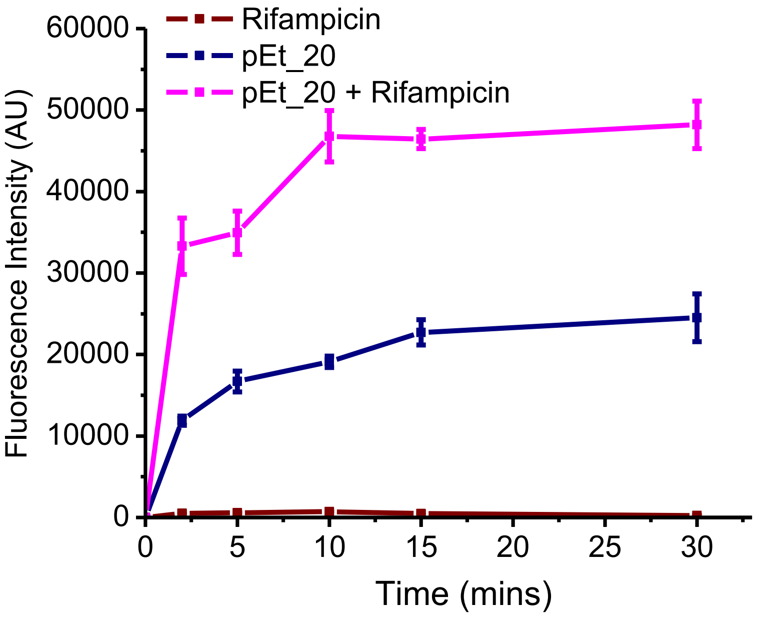

Figure S6. Increase in intracellular ROS of rifampicin-resistant *A. baumannii* BAA-1709 mutant. Fluorescence intensity analysis of intracellular ROS probe CellRox Green in rifampicin-resistant *A. baumannii* BAA-1709 mutant (~10^7 CFU/mL) after treatment with rifampicin (0.50 μg/mL), pEt_20 (7.8 μg/mL) and their combination (pEt_20: 7.8 μg/mL; rifampicin: 0.50 μg/mL) over various periods of time. The results showed that the combination significantly enhanced intracellular ROS generation. pEt_20 translocated bacterial membrane followed by binding of cytosolic proteins or genes, facilitating ROS generation and thus killing the bacteria.

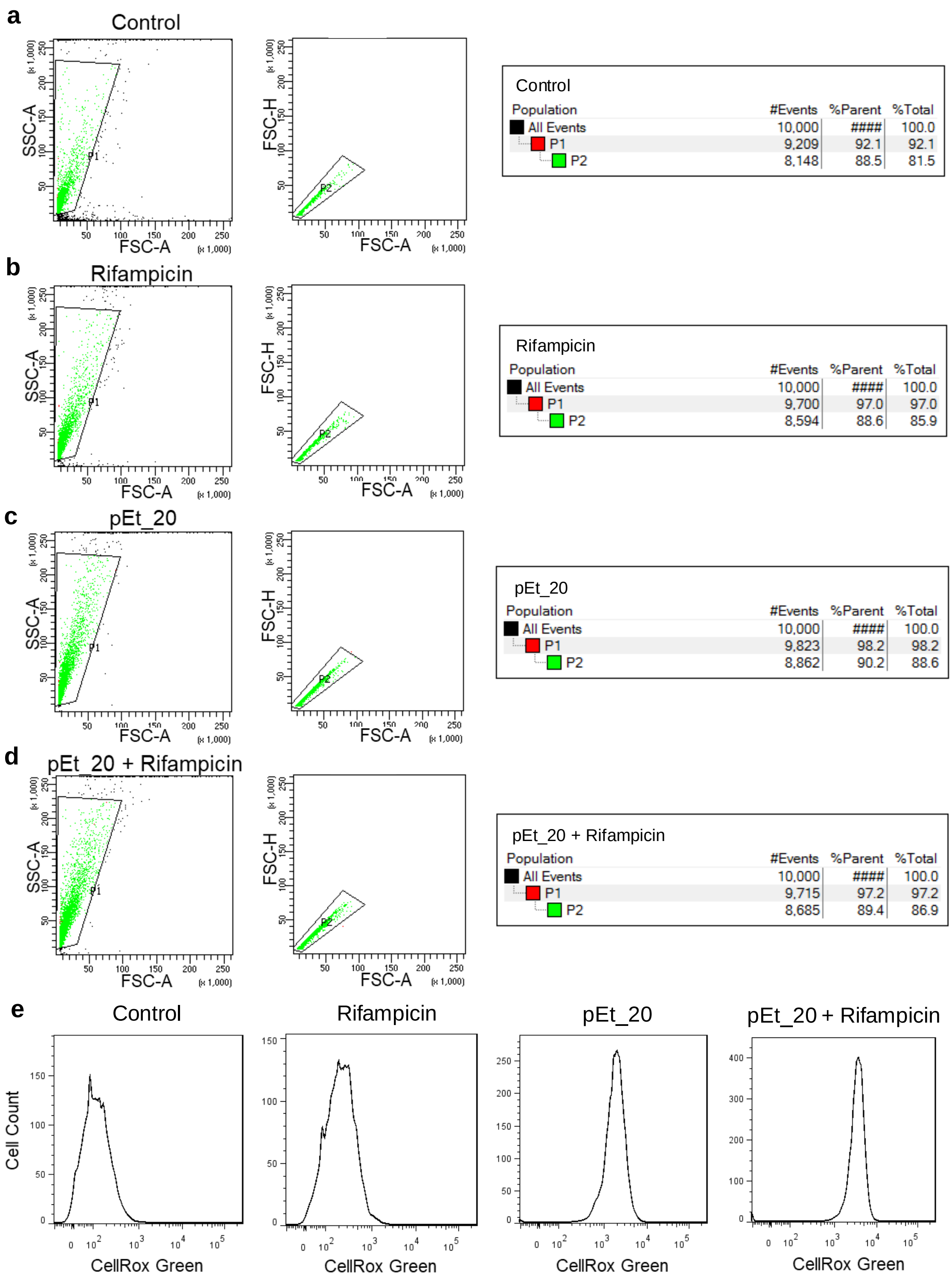

Figure S7. (for Figure 5f) The combination of pEt_20 and rifampicin significantly enhances intracellular ROS generation as indicated by flow cytometry results. **a-d** Gating strategy used for flow cytometry analysis of the intracellular probe CellRox Green-treated *A. baumannii* BAA-1709 cells. The P1 gating was to exclude the dead cells, which is located in the bottom left corner in FCS/SSC plots; The P2 gating was to choose the single cells in FSC-A/FSC-H plots; 10,000 cells were counted for each sample; **e** Individual flow cytometry histograms of CellRox Green-treated *A. baumannii* BAA-1709 cells after treatment with rifampicin (0.50 µg/mL), pEt_20 (7.8 µg/mL) and their combination (pEt_20: 7.8 µg/mL; rifampicin: 0.50 µg/mL) for 5 min.

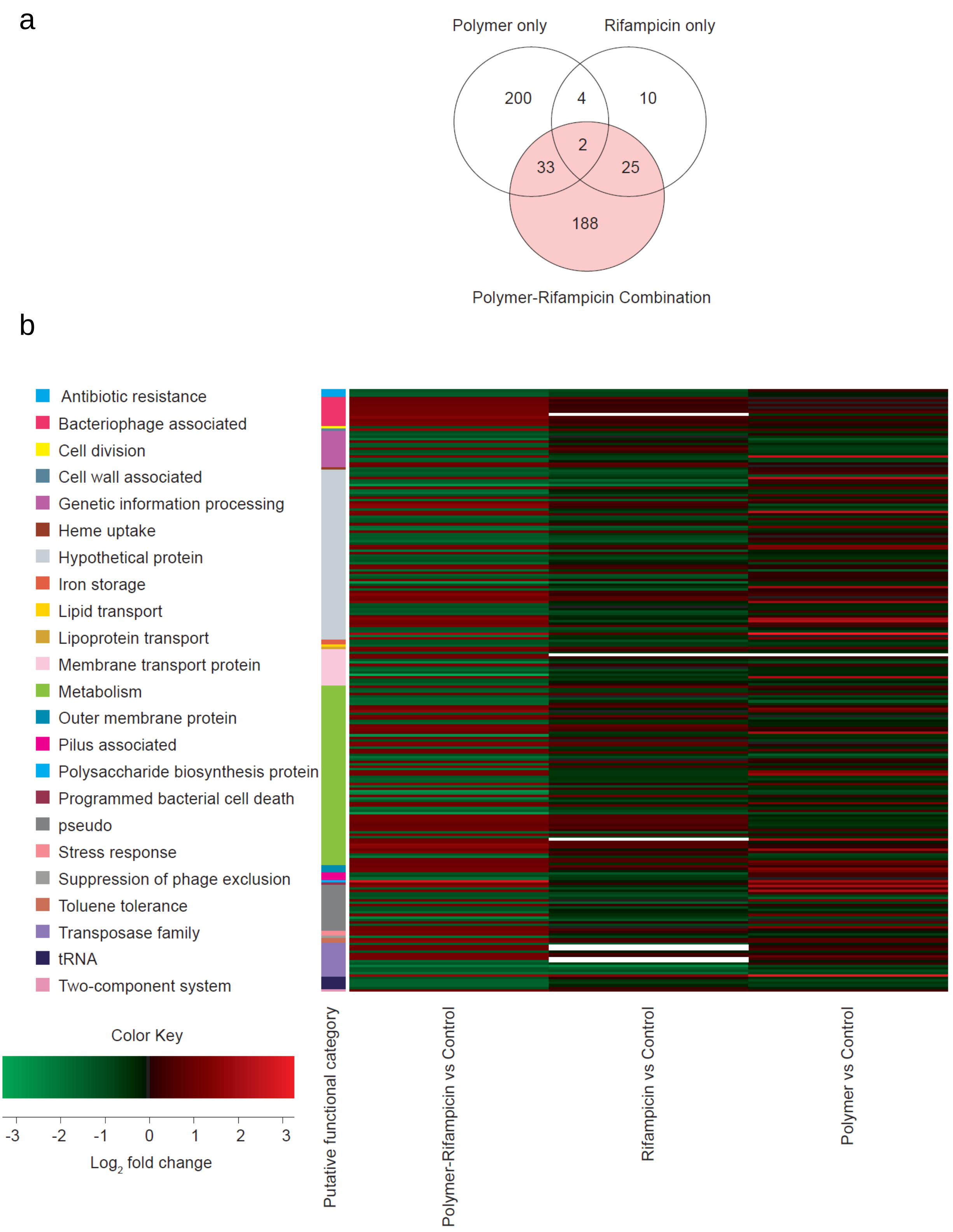

Figure S8. RNA-seq based comparative transcriptomics and functional annotation across different treatment conditions. **a** A venn diagram showing the number of differentially regulated genes upon the treatment of pEt_20, rifampicin or pEt_20/rifampicin combination, relative to untreated controls; Pink colored circle highlights the pEt_20/rifampicin combination treatment condition; **b** Heatmap depicts differentially expressed genes that are significant in "Polymer-Rifampicin" treatment condition in contrast to "Polymer only" and "Rifampicin only" treatments wherein the same genes from the latter might have not been

statistically significant. Gradient of green to red was used to depict intensity of down and up regulation in terms of log2 fold change of treatment conditions relative to control. Intermittent white lines in the heatmap denote genes that fell below coverage cut off for RNA-seq reads, therefore differential expression is undetermined. Colored bar panel on the left of heatmap refers to manually assigned putative functional categories (colored boxes and the legend text) of the corresponding genes in the heatmap.

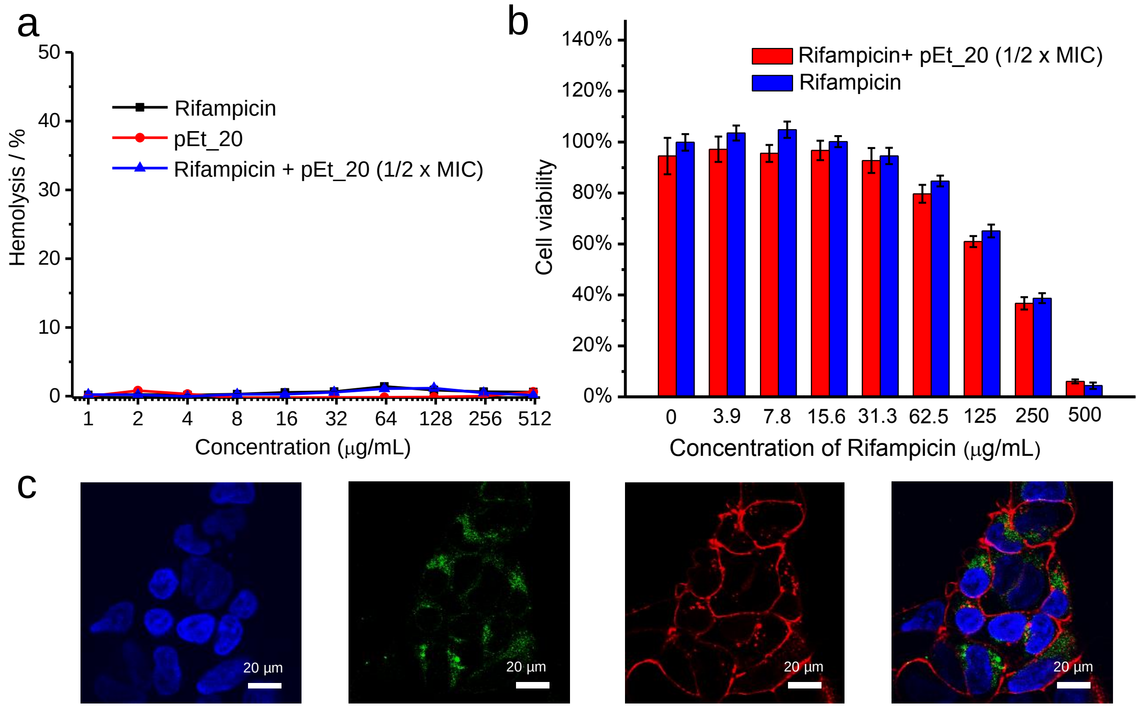

Figure S9. Combination with pEt_20 does not increase cytotoxicity of rifampicin to mammalian cells. **a** Hemolysis level of rifampicin, pEt_20 and their combination at different concentrations; All treatments did not cause hemolysis up to 500 μg/mL; **b** Viability of human embryonic kidney cells (HEK293T) after 24 h-treatment with rifampicin or pEt_20/rifampicin combination. pEt_20 did not affect cytotoxicity profile of rifampicin. At 31.3 μg/mL or below, there was no cytotoxicity of rifampicin observed; **c** The confocal microscopic results indicated that the polymer (pEt_20, 7.8 μg/mL) was localized in cytoplasma instead of nucleus. Treatment time: 1 h.

a

Treatment	ALT (U/L)	AST (U/L)	Creatinine μmol/L	Urea nitrogen mmol/L	Sodium ion mmol/L	Potassium ion mmol/L
PBS	37.3±9.1	92.8±34.3	<9.0	7.6±0.8	147.2±1.7	4.0±0.4
Rifampicin (2 days)	34.8±8.7	79.3±15.4	<9.0	7.6±0.6	146.7±1.2	4.1±0.2
Rifampicin + pEt_20 (2 days)	30.3±9.2	77.0±16.8	<9.0	6.8±0.4	147.5±0.5	4.3±0.2
Rifampicin (14 days)	32.6±7.1	72.4±12.1	<9.0	7.2±0.4	148.4±1.3	3.8±0.3
Rifampicin + pEt_20 (14 days)	25.7±7.3	77.3±18.1	<9.0	6.7 ±0.4	147.3±1.6	4.0±0.2

b

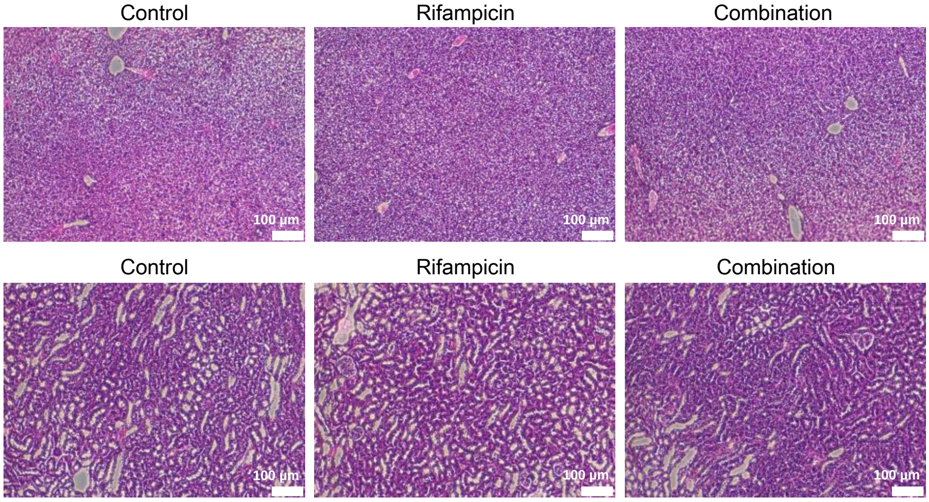

d

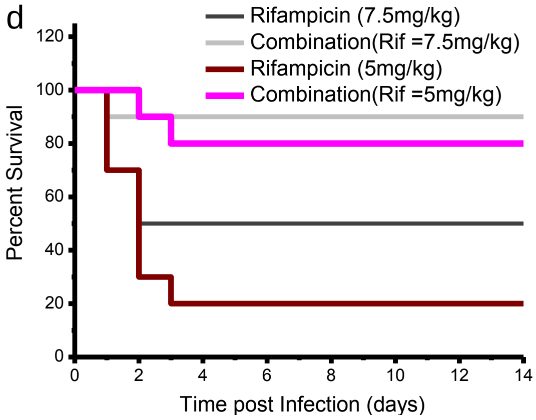

Figure S10. *In vivo* biocompatibility of pEt_20/rifampicin combination and antibacterial efficacy of pEt_20/rifampicin combination at different rifampicin doses. **a** Serum biochemistry analysis; Compared to PBS, rifampicin and pEt_20/rifampicin combination did not alter liver and kidney functions nor affect sodium ion and potassium ion concentrations ($p > 0.05$), indicating that rifampicin or the combination did not induce *in vivo* toxicity. Microscopic images of hematoxylin and eosin (H&E) - stained liver (**b**) and kidney (**c**) at 14 days post treatment with rifampicin (5.0 mg/kg) or the combination of rifampicin (5.0 mg/kg) and pEt_20 (2.0 mg/kg); The insignificant difference of tissue structures between pEt_20/rifampicin combination-treated mice and untreated or rifampicin-treated mice indicated that the combination did not induce hepatotoxicity and nephrotoxicity; **d** An increased rifampicin dose led to higher survival rate (n=10).

Table S1. The polymer pEt_20 sensitized MCR-1 positive colistin-resistant *E. coli* strains to colistimethate sodium treatment. Polymer concentration: 0.5×MIC (7.8 μg/mL). The polymer reversed colistin resistance phenotype, reducing its MIC from 15.6-31.3 μg/mL to 0.49 μg/mL. It also reduced MBC of colistimethate sodium by 8-fold from 15.6 and 31.3 μg/mL to 1.95 and 3.91 μg/mL, respectively. MIC and MBC data is representative of three biological replicates.

Bacteria	MIC of colistin (μg/mL)		MBC of colistin (μg/mL)	
	w/o polymer	With polymer*	w/o polymer	With polymer*
E. coli - 1	31.3	0.49	31.3	3.9
E. coli - 2	15.6	0.49	15.6	1.95

Table S2. The polymer pEt_20 repurposed rifampicin derivatives (rifaximin and rifabutin) as potent antibiotics against *A. baumannii* (BAA-1709). The polymer at 0.5×MIC (i.e. 7.8 µg/mL) reduced MIC of rifampicin derivatives (rifaximin and rifabutin) by 512-fold. MIC data is representative of three biological replicates.

Antibiotic	MIC (µg/mL)	
	w/o polymer	With polymer (fold reduction)
Rifaximin	3.91	0.0076 (512)
Rifabutin	7.81	0.015 (512)

Table S3. Effect of various compounds including cationic polycarbonate with quaternary ammonium (Qua_20), polyarginine with 10 amino acids (R_{10}), colistin sulfate, pEt_10, pEt_20 and pEt_40 on MIC of rifampicin against *A. baumannii* (BAA-1709). Use of guanidinium-functionalized polycarbonates (pEt_10, pEt_20 and pEt_40) potentiated rifampicin more effectively than the rest compounds, reducing rifampicin MIC by 2048 to 4096-fold.

Compounds	Rifampicin MIC (fold reduction) in the presence of different compounds at 0.5×MIC (μg/mL)
Qua_20 (MIC: 15.6 μg/mL)	0.98 (4)
R10 (MIC: 31.3 μg/mL)	0.98 (4)
Colistin (MIC: 1.0 μg/mL)	0.244 (16)
pEt_10 (MIC: 15.6 μg/mL)	0.0019 (2048)
pEt_20 (MIC: 15.6 μg/mL)	0.0019 (2048)
pEt_40 (MIC: 15.6 μg/mL)	0.00095 (4096)

Table S4. RNA-seq on *A. baumannii* BAA-1709 cells treated with rifampin for 5 min, relative to untreated control cells. (In the word file, please double click the table below to see the full list).

Locus-tag	Gene name	Product description	logFC	logCPM	PValue
ABSDF3397	NA	pseudo	2.75038382	2.639199	5.54E-05
ABSDF1359	NA	IS982 family transposase	1.49153608	4.452744	8.42E-05
ABSDF3602	NA	IS982 family transposase	1.32775427	3.555293	0.016632479
ABSDF3045	NA	pseudo	1.08966404	3.493362	0.001096222
ABSDF0431	rpmC	50S ribosomal protein L29	1.01951977	11.84988	1.02E-16
ABSDF0254	NA	pseudo	1.00522482	4.527912	0.001514906
ABSDF3597	hpd	4-hydroxyphenylpyruvate dioxyge	-1.007257	10.66986	8.01E-28
ABSDF1992	NA	hypothetical protein	-1.0134616	7.82507	5.98E-30
ABSDF2122	NA	hypothetical protein	-1.0149208	7.965829	1.91E-28
ABSDF3013	NA	hydrolase	-1.0285445	9.276849	7.34E-26
ABSDF0522	NA	pseudo	-1.0382909	7.425346	1.97E-30
ABSDF3489	NA	hypothetical protein	-1.0396111	10.95793	4.61E-27
ABSDF2566	NA	hypothetical protein	-1.0400809	8.222832	1.36E-24
ABSDF1698	NA	signal peptide	-1.0616396	3.469103	3.43E-07
ABSDF1830	NA	hypothetical protein	-1.0628058	5.313994	8.45E-13
ABSDF0317	bfrB	bacterioferritin	-1.0873115	8.604489	1.39E-32
ABSDF2565	NA	hypothetical protein	-1.100315	10.79073	3.80E-35
ABSDF2228	NA	hypothetical protein	-1.1372366	7.201906	8.09E-33
ABSDF2429	NA	pseudo	-1.1417021	2.668859	0.00030075
ABSDF2655	otsB	trehalose-6-phosphate phophatas	-1.148711	4.582244	4.56E-08
ABSDF3026	NA	hypothetical protein	-1.1621485	5.919265	1.55E-22
ABSDF1910	NA	methyltransferase	-1.1764917	9.557042	9.10E-16
ABSDF2192	NA	IS5 family transposase	-1.1984725	2.945334	1.75E-07
ABSDF1165	NA	IS982 family transposase	-1.2000702	1.257168	6.25E-05
ABSDF2834	NA	pilus assembly protein FilB	-1.2092649	7.225845	1.32E-22
ABSDF2046	aro	phospho-2-dehydro-3-deoxyhept	-1.2138201	8.438065	4.98E-36

Table S5. RNA-seq on *A. baumannii* BAA-1709 cells treated with pEt_20 for 5 min, relative to untreated control cells. (In the word file, please double click the table below to see the full list).

Locus-tag	Gene name	Product description	logFC	logCPM	PValue
ABSDF3350	NA	signal peptide	3.24886	7.82486274	1.53E-193
ABSDF1359	NA	IS982 family transposase	3.061063	5.97541646	1.92E-07
ABSDF2215	NA	hypothetical protein	2.555296	4.97783899	4.95E-55
ABSDF3326	NA	thymidylate synthase	2.550949	6.87162395	3.29E-145
ABSDF3325	NA	hypothetical protein	2.451616	3.22231838	3.17E-21
ABSDF1200	NA	hypothetical protein	2.446091	8.75424176	3.35E-213
ABSDF2041	fadH	pseudo	2.358722	4.82445168	1.92E-55
ABSDF0533	NA	hypothetical protein	2.353601	3.95914602	1.32E-38
ABSDF2313	NA	hypothetical protein	2.34164	5.32799649	1.18E-73
ABSDF3324	NA	hypothetical protein	2.296721	6.82141566	4.40E-91
ABSDF2042	fadH	pseudo	2.280224	6.05410291	5.05E-95
ABSDF3602	NA	IS982 family transposase	2.272546	4.53020397	0.000131123
ABSDF3329	NA	hypothetical protein	2.263146	2.17115015	2.38E-19
ABSDF3353	NA	hypothetical protein	2.142744	3.56947447	1.83E-26
ABSDF1207	NA	membrane protein branched-cl	2.116231	3.40345873	3.12E-24
ABSDF1206	NA	pseudo	2.104189	2.46675289	7.93E-21
ABSDF0637	NA	pseudo	2.09452	2.78157203	6.01E-20
ABSDF2040	fadH	pseudo	2.089076	4.57773832	1.95E-32
ABSDF3330	NA	ATP-binding	2.086455	4.44557645	5.55E-40
ABSDF1467	NA	IS982 family transposase	2.083449	3.92440752	0.002317
ABSDF0727	NA	SAM-dependent methyltransfera	2.074423	2.66099594	1.21E-19
ABSDF3327	NA	hypothetical protein	2.016346	5.92970748	3.13E-69
ABSDF0049	NA	oxidoreductase	2.001574	6.84986521	1.06E-93
ABSDF0862	NA	pseudo	1.955488	3.18702908	3.02E-19
ABSDF0075	NA	polysaccharide biosynthesis pro	1.943847	3.13174817	1.00E-18
ABSDF1269	NA	hypothetical protein	1.937373	7.01237651	5.31E-107
ABSDF2043	fadH	pseudo	1.935158	6.19161655	6.69E-71

Table S6. RNA-seq on *A. baumannii* BAA-1709 cells treated with pEt_20/rifampicin combination for 5 min, relative to untreated control cells. (In the word file, please double click the table below to see the full list).

Locus-tag	Gene name	Product description	logFC	logCPM	PValue
ABSDF0075	NA	polysaccharide biosynthesis protein	2.786488	3.63568484	2.66E-27
ABSDF3350	NA	signal peptide	1.975524	6.57871484	8.11E-64
ABSDF1359	NA	IS982 family transposase	1.779427	4.72558742	0.0207515
ABSDF1861	NA	acyl-CoA dehydrogenase	1.692394	5.49847857	3.65E-47
ABSDF0907	pal	peptidoglycan-associated lipoprotein	1.687457	9.36354221	4.31E-92
ABSDF1939	NA	hypothetical protein	1.686021	4.48551059	1.99E-27
ABSDF0779	sucD	succinyl-CoA synthetase subunit alpha	1.685146	12.0426669	6.03E-93
ABSDF1269	NA	hypothetical protein	1.625216	6.61439267	1.39E-75
ABSDF1938	NA	hypothetical protein	1.618615	7.48865192	5.37E-85
ABSDF1940	NA	homoserine dehydrogenase HDH	1.568337	3.8455438	1.27E-18
ABSDF3327	NA	hypothetical protein	1.537554	5.39889345	3.95E-38
ABSDF0397	NA	toluene-tolerance protein Ttg2E	1.526315	8.01998117	2.94E-84
ABSDF0778	sucC	succinyl-CoA synthetase subunit beta	1.490374	12.1985951	2.56E-84
ABSDF2040	fadH	pseudo	1.489227	3.9438923	1.81E-15
ABSDF1754	NA	phage-like protein	1.489131	2.46309211	1.78E-10
ABSDF1771	NA	hypothetical protein	1.472403	1.91186459	1.90E-09
ABSDF0365	NA	signal peptide	1.450386	5.83995619	3.73E-48
ABSDF0082	galE	UDP-glucose 4-epimerase	1.43173	8.57521825	2.58E-67
ABSDF3325	NA	hypothetical protein	1.397009	2.20525393	1.71E-08
ABSDF0777	lpd	dihydrolipoamide dehydrogenase	1.391102	12.3110094	1.54E-63
ABSDF0997	NA	phage-like protein	1.347177	2.04083423	4.94E-07
ABSDF0544	NA	hypothetical protein	1.340812	5.19923806	5.95E-27
ABSDF1122	purB	adenylosuccinate lyase	1.338083	9.35957961	8.30E-74
ABSDF0882	lolD	outer membrane lipoproteins ABC trans	1.326679	7.13810412	9.56E-76
ABSDF0533	NA	hypothetical protein	1.323273	2.98011811	6.44E-11
ABSDF1010	NA	hypothetical protein	1.317878	2.92379812	1.57E-07
ABSDF2032	eno	phosphopyruvate hydratase	1.315891	10.1433587	3.71E-44

Table S7. Differentially regulated genes upon the treatment with pEt_20/rifampicin combination but not with rifampicin (Rif) treatment or polymer treatment alone. (In the word file, please double click the table below to see the full list).

Locus_tag	Protein name	pEt_20	Rif vs. Cc	pEt_20+Rif v	Putative functional annotation
ABSDF0011	RND type efflux pump in	0.243	-0.8579	-1.138104	Antibiotic resistance
ABSDF0008	RND type efflux pump in	-0.1	-0.7188	-1.297772	Antibiotic resistance
ABSDF0010	RND type efflux pump in	-0.06	-0.704	-1.3694	Antibiotic resistance
ABSDF2476	bacteriophage protein	0.042	0.77761	1.0105386	Bacteriophage associated
ABSDF2471	bacteriophage protein	0.332	0.17653	1.0023388	Bacteriophage associated
ABSDF2473	bacteriophage protein	0.044	0.44359	1.0789438	Bacteriophage associated
ABSDF2497	bacteriophage protein	0.221	0.14406	1.0963925	Bacteriophage associated
ABSDF2469	bacteriophage protein	0.004	0.25672	1.1152003	Bacteriophage associated
ABSDF1029	bacteriophage protein p	0.246	0.32149	1.1491374	Bacteriophage associated
ABSDF2502	bacteriophage regulatory	0.706	0.30696	1.1470215	Bacteriophage associated
ABSDF2608	phage-like protein	-0.57	NA	1.2641299	Bacteriophage associated
ABSDF1754	phage-like protein	0.316	0.50618	1.489131	Bacteriophage associated
ABSDF1785	phage-like protein	-0.2	0.26123	1.0473229	Bacteriophage associated
ABSDF1004	phage-like protein	0.224	0.14779	1.2692441	Bacteriophage associated
ABSDF0997	phage-like protein	0.069	0.52728	1.3471773	Bacteriophage associated
